# Altered salience network structure–function integration underlies the decline in cognitive flexibility during aging

**DOI:** 10.1371/journal.pbio.3003738

**Published:** 2026-04-13

**Authors:** Xing Qian, Wan Lin Yue, Kwun Kei Ng, Ruth L. F. Leong, Fang Ji, Narayanaswamy Venketasubramanian, Saima Hilal, Christopher Chen, Michael W. L. Chee, Dani S. Bassett, Juan Helen Zhou

**Affiliations:** 1 Centre for Sleep and Cognition & Centre for Translational Magnetic Resonance Research, Yong Loo Lin School of Medicine, National University of Singapore, Singapore, Singapore; 2 Integrative Sciences and Engineering Programme, NUS Graduate School, National University of Singapore, Singapore, Singapore; 3 Raffles Neuroscience Centre, Raffles Hospital, Singapore, Singapore; 4 Memory Aging and Cognition Centre, Department of Pharmacology, Yong Loo Lin School of Medicine, National University of Singapore, Singapore, Singapore; 5 Departments of Physics & Astronomy, Bioengineering, Electrical & Systems Engineering, Neurology, and Psychiatry, University of Pennsylvania, Philadelphia, Pennsylvania, United States of America; 6 Department of Electrical and Computer Engineering, National University of Singapore, Singapore, Singapore; 7 Department of Medicine, Healthy Longevity & Human Potential TRP, Yong Loo Lin School of Medicine, National University of Singapore, Singapore, Singapore; Sungkyunkwan University, KOREA, REPUBLIC OF

## Abstract

Cognitive flexibility supports efficient switching between mental sets and contributes to the preservation of general cognition in aging. It relies on the integration between brain functional dynamics and structural architecture. However, how this structure–function integration changes with age and contributes to cognitive flexibility decline in older adults remains unclear. In this study, we investigated longitudinal aging-related changes in multimodal structure–function integration, quantified as functional signal alignment (i.e., coupling) versus liberality (i.e., decoupling) relative to individual structural connectomes, which represent distinct spectral components, and tested their longitudinal associations with cognitive flexibility. Resting-state fMRI signals were decomposed based on diffusion MRI–derived structural networks using a graph signal processing framework. We focused on subnetworks within three core large-scale cognitive systems: the executive control network (ECN), default mode network (DMN), and salience network (SN). Across two independent datasets, the task-positive SN-A subnetwork, which includes core SN regions such as the anterior insula and dorsal anterior cingulate cortex, exhibited decreased coupling and increased decoupling with aging. Importantly, these changes were associated with a greater decline in cognitive flexibility (measured by the Trail Making Test and Color Trails Test) over time. In contrast, task-negative DMN-A (centered in the medial prefrontal and posterior cingulate cortex) showed aging-related changes in the opposite direction, with increased coupling and decreased decoupling over time. Together, these findings reveal network-specific trajectories of intrinsic structure–function integration in normal aging and indicate that preserved structure–function integration within the SN may be particularly important for maintaining cognitive flexibility in older adults.

## Abstract 2

Aging alters how the brain’s structural wiring constrains its functional activity. This study reveals how changes in this balance, particularly within the salience network, predict declines in cognitive flexibility over time, highlighting a key neural mechanism supporting cognitive resilience in later life.

## Introduction

Cognitive flexibility is a core executive function that enables efficient switching between different perspectives, goals, or tasks, thereby supporting adaptive thinking and behavior in response to changing circumstances [1–3]. It relies on the coordinated operation of multiple executive processes, including salience detection, attention, working memory, inhibition, and cognitive switching [4,5]. In older adults, preserved cognitive flexibility is particularly important for maintaining independence and quality of life, whereas declines in flexibility can contribute to broader age-related cognitive difficulties and reduced daily functioning [6–9]. Understanding the neural mechanisms that support cognitive flexibility is therefore essential for developing effective strategies to mitigate cognitive decline and promote healthy aging.

Normal aging is accompanied by widespread alterations in brain structure and function, including degeneration of white matter pathways [10–12], reductions in grey matter integrity [13–19], and changes in functional brain activity [20–25]. At the network level, older adults typically show reduced within-network specialization and diminished segregation between functional networks, particularly within the executive control network (ECN), default mode network (DMN), and salience network (SN) [26–30]. These networks are critically involved in higher-order cognitive processes that are known to decline with age [26,29,31,32]. Specifically, the ECN supports cognitive control functions and goal-directed behavior [32,33], the DMN is implicated in internally directed cognition, including self-referential thought and autobiographical memory [34], and the SN plays a central role in detecting salient stimuli and orchestrating dynamic switching between the ECN and DMN [33,35]. This latter function enables the SN to mediate the shift between externally oriented attention and internally focused mental states, a mechanism that becomes increasingly important for cognitive flexibility in aging.

In parallel, diffusion MRI studies consistently report reduced structural connectivity (SC) and network efficiency with aging, with decreases both within and between network modules [36–39], and converging evidence suggests that age-related functional connectivity (FC) and SC alterations show partially overlapping spatial patterns and are related to one another [26,27,40,41]. Importantly, age-related disruptions in both functional and SC within and between the core cognitive networks are associated with poorer cognitive performance, suggesting that coordinated structure–function alterations may contribute to cognitive decline in aging [11,14,26,29,42–44]. Because neuroanatomical connectivity constrains functional interactions, the relationship between SC and FC has been widely studied as an index of structure–function coupling [45–53]. It has been widely examined as a marker of coordinated brain organization and its disruption in aging and disease [27,41,54–59].

However, the simple correlation between SC and FC offers a mechanistic, yet incomplete, explanation of brain function [48,60,61]. Recent graph signal processing approaches instead quantify structure–function integration by directly characterizing how functional signals are constrained by, or deviate from, the underlying structural connectome. Using a graph Fourier framework, BOLD activity can be decomposed into structurally aligned versus structurally liberal components, which represent distinct spectral components [62,63]. Specifically, alignment reflects stronger structural constraints on intrinsic functional dynamics (i.e., higher coupling), whereas liberality reflects greater deviations from structural architecture (i.e., higher decoupling). Importantly, these indices differ from conventional SC–FC coupling measures because they are derived from graph spectral decomposition of BOLD signals with respect to the structural connectome, rather than from correlations between SC and FC. To improve accessibility, we refer to alignment and liberality as coupling and decoupling, respectively, while noting that these terms are defined within the present graph-spectral framework and are distinct from correlation-based coupling/decoupling metrics. Notably, in young adults performing a cognitive switching task, better performance was associated with reduced signal liberality/decoupling, suggesting that tighter structural constraints may support cognitive flexibility [62]. This framework therefore offers a promising avenue for testing whether age-related alterations in intrinsic structure–function integration contribute to longitudinal decline in cognitive flexibility [64–68], which has been linked to both structural and functional brain changes in older adults [69–71]. In parallel, prior work has demonstrated that cognitive flexibility across the life span is supported by large-scale brain dynamics [72]. However, how the functional dynamics constrained by the underlying structural connectome change in aging remains largely unexplored.

Accordingly, the present study examined longitudinal changes in intrinsic structure–function integration (coupling and decoupling) at the network level in normal aging and tested whether these changes predict longitudinal decline in cognitive flexibility [measured by out-of-scanner Trail Making Test (TMT) and Color Trails Test (CTT)] in older adults. Since most prior structure–function integration studies in aging are cross-sectional, making it difficult to distinguish true within-person change from cohort effects, our longitudinal approach allows us to quantify within-individual trajectories of structure–function coupling/decoupling. Particularly, we assessed intrinsic structure–function integration from resting-state fMRI, providing a task-independent characterization of aging-related changes that complements prior task-based work. We focused on the subnetworks of ECN, DMN, and SN because they form core large-scale systems supporting higher-order cognitive function including cognitive flexibility and executive control, show robust age-related alterations in functional segregation and SC, and the SN in particular coordinates switching between ECN and DMN [26–30]. We hypothesized that functional signals would become progressively less coupled (or more decoupled) with respect to the underlying neuroanatomical architecture over time, and that greater deviations from normative integration would be associated with steeper cognitive flexibility decline.

## Methods

### Participants

Two independent datasets comprising healthy older adults were used to investigate aging-related changes in structure–function integration. The primary dataset was drawn from the neuroimaging subset of the Singapore-Longitudinal Aging Brain Study (S-LABS) [73]. Participants were included only if they had at least two time points of neuroimaging data that passed quality control procedures (see Image processing), with inter-scan intervals ranging from 18 to 24 months over a 5-year period. To ensure a healthy aging cohort, participants were excluded if they met any of the following criteria at any time point: (1) Mini-Mental State Examination score <26; (2) modified-Geriatric Depression Screening Scale score ≥9; (3) history of significant vascular events; (4) history of malignant neoplasia; (5) history of organ failure (heart, lung, liver, or kidney); (6) thyroid disease (active or inadequately treated); (7) existing neurological or psychiatric conditions; or (8) history of head trauma accompanied by loss of consciousness. These exclusion criteria were consistent with previous studies using the same dataset [29,42,73]. The final sample consisted of 54 healthy older adults (24 females; age range: 59–82 years; mean baseline age = 66.7 ± 4.9 years). Of these, 35 participants had data from two time points, while 19 participants had data from three time points.

A second dataset of healthy older adults, described in a previously published study [74,75], was used for independent replication and validation of the primary findings. Participants were excluded based on the following criteria: (1) history of stroke; (2) MRI evidence of cerebrovascular disease (defined by MRI markers: presence of cortical infarcts and/or ≥ 2 lacunes and/or confluent white matter lesions in two brain regions with Age Related White Matter Changes score ≥8); (3) diagnosis of cognitive impairment from review of clinical measures and neuropsychological assessments; and (4) less than two time points of neuroimaging data that passed quality control criteria. The final validation sample included 39 healthy older adults (29 females; age range: 62–80 years; mean baseline age = 68.9 ± 5.1 years). Of these, 27 participants had data from two time points, and 12 participants had data from three time points (mean interval = 2.7 ± 1.0 years).

Ethical approval for the two studies was obtained from the Institutional Review Board of the National University of Singapore (H-17-077) and the Domain Specific Review Board of the National Healthcare Group, Singapore (2010/00017), respectively. All participants provided written informed consent prior to participation. The study was conducted in accordance with the ethical principles of the Declaration of Helsinki.

### Neuropsychological assessments

To assess cognitive flexibility in the main dataset, a subset of 52 participants (24 females, mean baseline age = 66.7 ± 4.9 years) completed Parts A and B of the TMT [14,73,76,77] within three months of each neuroimaging session. All participants had at least two time points with both neuroimaging and TMT data (mean interval = 2.6 ± 1.0 years). In brief, TMT A required participants to sequentially connect the numbers 1–25, which were randomly distributed across a sheet of paper. TMT B introduced a cognitive switching component by requiring participants to alternate between numbers (1–13) and letters (A–L) in ascending order (e.g., 1–A–2–B…). According to the concept of global switching cost, defined as the increase in response time for switching versus non-switching tasks [66,67], we calculated dTMT, the difference in completion time between TMT B and TMT A (dTMT = TMT B − TMT A), as a measure of cognitive flexibility. These dTMT values were standardized into T scores by first z-scoring and then transforming them to a distribution with a mean of 50 and a standard deviation of 10, for use in statistical analyses.

Cognitive flexibility in the validation dataset was assessed using Parts A and B of the CTT [78–80], which was developed as an alternative to TMT. CTT A, which is structurally identical to TMT A, required participants to sequentially connect numbers 1–25. CTT B involved the same numerical sequence but added a switching demand by presenting the numbers in two different colors, requiring participants to alternate between colors while maintaining numeric order. As with the main dataset, the difference in completion time between CTT B and CTT A (dCTT = CTT B − CTT A) was used to index cognitive flexibility. These dCTT values were also converted to T scores and used in the statistical analyses.

### Image acquisition

Participants from both the main and validation datasets were scanned using a 3T Siemens Tim Trio system. For the main dataset, high-resolution T1-weighted structural scans were acquired using a magnetization prepared rapid acquisition gradient echo (MPRAGE) sequence with the following imaging parameters: 192 continuous sagittal slices, TR = 2,300 ms, TE = 2.98 ms, TI = 900 ms, flip angle = 9°, FOV = 256 × 256 mm^2^, voxel size = 1.0 × 1.0 × 1.0 mm^3^. High-resolution T2-weighted structural scans were also acquired with the following imaging parameters: 192 continuous sagittal slices, TR = 3,200 ms, TE = 448.0 ms, TI = 1,800 ms, flip angle = 0°, FOV = 256 × 256 mm^2^, voxel size = 1.0 × 1.0 × 1.0 mm^3^. Resting state fMRI scans (8 min) were acquired for the main dataset with participants fixating on a cross presented in the center of the screen (36 continuous axial slices, TR = 2,000 ms, TE = 30 ms, flip angle = 90°, FOV = 192 × 192 mm^2^, voxel size = 3.0 × 3.0 × 3.0 mm^3^). Diffusion-weighted MRI (dMRI) scans for the main dataset were acquired using a single-shot spin echo planar imaging sequence with the following imaging parameters: 54 continuous axial slices, TR = 9 600 ms, TE = 107 ms, FOV = 256 × 256 mm^2^, voxel size = 2.0 × 2.0 × 2.0 mm^3^, 30 non-collinear diffusion gradient directions at *b* = 1,000 s/mm^2^, 6 volumes of *b* = 0 s/mm^2^.

In the validation dataset, high-resolution T1-weighted structural scans were acquired using the same MPRAGE sequence as the main dataset (except TE = 1.9s). A shorter resting state fMRI scan (5 min) with the same fixation instruction was collected (48 continuous axial slices, TR = 2,300 ms, TE = 25 ms, flip angle = 90°, FOV = 192 × 192 mm^2^, voxel size = 3.0 × 3.0 × 3.0 mm^3^). The dMRI scans were acquired with a different set of imaging parameters (48 continuous axial slices, TR = 6,800 ms, TE = 85 ms, FOV = 256 × 256 mm^2^, voxel size = 3.0 × 3.0 × 3.0 mm^3^, 61 non-collinear diffusion gradient directions at *b* = 1,150 s/mm^2^, 7 volumes of *b* = 0 s/mm^2^). For the validation dataset, fluid attenuated inversion recovery scans were also collected for assessment of cerebrovascular disease markers (48 continuous axial slices, TR = 9,000 ms, TE = 82 ms, flip angle = 180°, FOV = 256 × 256 mm^2^, voxel size = 1.0 × 1.0 × 3.0 mm^3^).

### Image processing

Structural and functional images from both the main and validation datasets were preprocessed using standard protocols implemented in FSL [81] and AFNI [82], following procedures established in prior studies [11,29,42]. In brief, preprocessing of T1 images involved reduction of image noise (SUSAN), skull stripping (Brain Extraction Tool), registration to the Montreal Neurological Institute (MNI) 152 standard space (linear and non-linear using FLIRT and FNIRT, respectively), and segmentation of the brain into grey matter, white matter, and cerebrospinal fluid (CSF).

Preprocessing of fMRI images involved removal of the first five volumes, correction for slice timing and motion, skull stripping, spatial smoothing (Gaussian kernel of 6 mm full-width half maximum), temporal band pass filtering (0.009–0.1 Hz), removing first and second order trends, co-registration with structural image (Boundary-Based Registration), non-linear registration to standard space (FNIRT), and nuisance signal regression (CSF, white matter, global signal and 6 motion parameters). Global signal regression (GSR) was applied in the main pipeline to reduce global shared fluctuations. To assess robustness, we repeated the key analyses using the same preprocessing pipeline without GSR.

Preprocessing of dMRI images involved correction of eddy current distortion and head motion through affine registration of dMRI images to the first *b* = 0 volume. Rotation of diffusion gradients was performed to compensate for motion. An additional distortion correction step using T2 images was performed for the main dataset. Fractional anisotropy (FA) images were obtained by voxel-wise fitting of a diffusion tensor model to the diffusion data (DTIFIT).

Quality control procedures were applied to exclude participants with excessive head motion. For fMRI, participants were excluded if maximum absolute displacement exceeded 4 mm or maximum framewise displacement exceeded 1 mm. For dMRI, participants were excluded if their maximum displacement relative to the first b = 0 volume exceeded 3 mm. Additionally, all fMRI and dMRI images were visually inspected for accurate co-registration with corresponding T1-weighted structural images.

### Structural network construction and BOLD time series extraction

**Fig 1:**
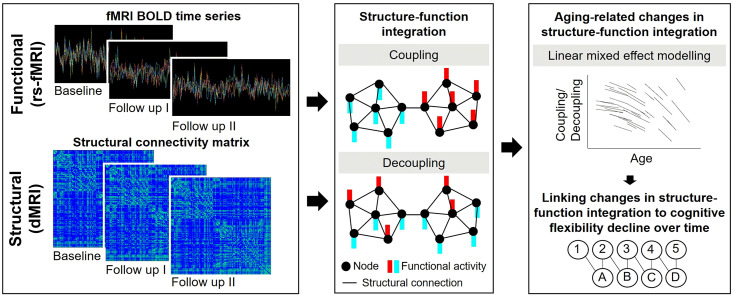
Overview of the study design. Neuroimaging data were collected at two to three time points per participant. Resting-state functional MRI (rs-fMRI) was used to extract time series of brain activity, while diffusion-weighted MRI (dMRI) was used to construct structural connectivity (SC) matrices. Using the graph Fourier transform, functional signals were decomposed into a portion that was aligned (coupled) with the underlying structural network (i.e., signals that vary smoothly across densely connected nodes), and another portion that was liberal (decoupled), deviating from the structural network (i.e., signals that vary sharply between densely connected nodes). In the illustration, SC between nodes (i.e., regions of interest [ROIs]) is shown as black edges, and the amplitude of BOLD fMRI signals at each ROI is represented by colored bars. Longitudinal changes in coupling and decoupling were modeled using linear mixed-effects models and evaluated in relation to cognitive flexibility decline over time.

An overview of the methodology used in this study is illustrated in Fig 1. Using a network parcellation of 126 regions of interest (ROIs), consisting of 114 cortical ROIs defined by Yeo and colleagues [83], which can be grouped into seven large-scale networks (DMN, ECN, SN, limbic network, dorsal attention network, somatomotor network and visual network), and 12 subcortical ROIs grouped into a subcortical network [84], BOLD signal time series was extracted for each ROI from the preprocessed fMRI of each individual. Given that the limbic network has been reported to exhibit the lowest test–retest reliability [85,86], its ROIs were excluded from further analysis. To assess the robustness of our findings, we repeated the analyses in the main dataset using an alternative brain parcellation with 416 ROIs, constructed by combining the Schaefer’s 400-parcel (17-network) cortical parcellation with the Tian’s 16-parcel subcortical atlas [87,88].

To construct the structural network from the preprocessed DTI data for each individual, the voxel-wise probabilistic distribution of fiber direction was first modeled using Bayesian estimation of diffusion parameters (bedpostX) [89]. Next, the predefined 122 ROIs (after removal of ROIs from the limbic network) from the same brain ROI parcellation template were used for probabilistic fiber tracking [90] in the Pipeline for Analyzing braiN Diffusion imAges (PANDA) toolbox [91]. One ROI with insufficient brain coverage (<95%) in the DTI images was dropped across participants (also removed from fMRI for consistency), resulting in a 121 ROI × 121 ROI SC matrix for each participant. Since the estimated probabilities between any two ROIs are different depending on the ROI from which the fiber tracks initiated, we averaged the two unidirectional connectivity probabilities as connection probability $P_{ij}$ for each pair of ROIs [36]. To account for differences in overall connection density and magnitude across participants (i.e., network cost), and to facilitate inter-subject comparability, the raw connection probabilities $P_{ij}$ between the *i*th ROI and *j*th ROI in the symmetrical matrix were log-transformed and normalized [56,92]. The final normalized weight $w_{ij}$ was calculated as:


$$w_{ij}=\;\frac{\text{log}\left(P_{ij}\right)-{\text{min}[\text{log}\left(P_{ij}\right)]}_{\text{1}<i\neq j<n}}{{\text{max}[\text{log}\left(P_{ij}\right)]}_{\text{1}<i\neq j<n}-{\text{min}[\text{log}\left(P_{ij}\right)]}_{\text{1}<i\neq j<n}},$$ (1)

where *n* is the number of ROIs. The resulting individual structural network served as the anatomical network upon which the resting-state fMRI signals from the same participant were projected and quantified for degree of coupling and decoupling.

### Derivation of structure–function coupling and decoupling

Measures of structure–function coupling and decoupling were derived following the graph signal processing framework proposed by Medaglia and colleagues [62]. For clarity and accessibility, we use the terms coupling and decoupling to refer to the alignment and liberality indices in the original study. Importantly, these measures should not be conflated with conventional structure–function coupling metrics based on SC–FC correlations, as they are computed via graph spectral decomposition of BOLD signals with respect to the structural connectome.

Specifically, eigenvector decomposition was performed on the symmetric structural network $A\in R^{n\times n}$, where *n* is the number of nodes (i.e., ROIs), to obtain a set of eigenvalues and corresponding eigenvectors:


$$A=V\Lambda V^{T},$$ (2)

where Λ denotes the diagonal matrix of eigenvalues, ordered as $\lambda_{\text{0}}\leq \lambda_{\text{1}}\leq \ldots \leq \lambda_{n-\text{1}}$, and $V=\overset{n-\text{1}}{\underset{k=\text{0}}{\left\{v_{k}\right\}}}$ is the matrix of associated eigenvectors. These eigenvectors form the spatial frequency basis of the graph A: “spatially rough” components (corresponding to negative eigenvalues) reflect highly connected nodes possess values of different signs (i.e., signal variation is abrupt across the network), whereas “spatially smooth” components indicate strongly connected nodes possess values of same signs (i.e., signal variation is coherent across the network).

A graph Fourier transform (GFT) was then applied to the fMRI BOLD time series $x\in R^{n}$, defined over brain ROIs, projecting the signals onto the structural eigenbasis:


$$\tilde{x}=V^{T}x={\left[{\tilde{x}}_{\text{0}},\;{\tilde{x}}_{\text{1}},\ldots ,{\tilde{x}}_{n-\text{1}}\right]}^{T}.$$ (3)

The original signal can be reconstructed as:


$$x=V\tilde{x}=\sum_{k=\text{0}}^{n-\text{1}}{\tilde{x}}_{k}v_{k}.$$ (4)

This transformation yields a spatial frequency-domain representation of the fMRI signal, where each GFT coefficient ${\tilde{x}}_{k}$ quantifies the contribution of a specific eigen vector $v_{k}$. The weighted “spatially smooth” components reflect the portion of functional signals aligned with the underlying structural network, whereas the weighted “spatially rough” components indicate the portion of functional signals deviating liberally from the structural network.

To extract alignment (coupling) and liberality (decoupling) measures which represent distinct spectral components, we applied graph filters to retain only the most structurally aligned (coupled) or liberal (decoupled) components. $K_{A}$ and $K_{L}$ denote the number of eigenvectors retained for the coupled and decoupled components, respectively. Rather than selecting a fixed number of graph spectral components or applying a spectrum dichotomization threshold as in prior studies [62,63], we determined the coupling and decoupling components using a variance-based criterion derived from the structural eigenvalue spectrum, which provides a principled way to define low- and high-frequency subspaces and is robust across parcellations with different ROI counts. In brief, for each group of the “spatially rough” and “spatially smooth” components, corresponding to negative and positive eigenvalues, respectively, we computed the total spectral energy by summing the absolute values of eigenvalues. The eigenvalues were then sorted in descending order within each group, and the cumulative sum was calculated. The value of $k_{A}$ (or $k_{L}$) was defined as the minimum number of top eigenvalues required to explain 95% of the total spectral energy within that group. This procedure was repeated for each time point, and the resulting $k_{A}$ (or $k_{L}$) values were averaged across time points within each subject to yield subject-specific values for $K_{A}$ and $K_{L}$. This approach allowed us to ensure that the selected eigenvectors represented the most informative smooth and rough components of the structural graph spectrum, while maintaining consistency across individuals and time points.

For implementation, we used the 95% variance threshold as the main analysis and repeated the procedure using 80% and 90% thresholds to assess robustness. For the main dataset, the average numbers of retained eigenvectors were: $K_{A}$= 7, 12, 16 and $K_{L}$ = 54, 66, 75; for the validation dataset, $K_{A}$ = 8, 13, 18 and $K_{L}$= 52, 65, 73 (corresponding to 80%, 90%, and 95% variance explained, respectively).

For each participant, the coupled and decoupled signal components were averaged across time and ROIs to produce static network-level measures of structure–function coupling and decoupling. In addition to averaging the coupled and decoupled signal components across time points to obtain static, network-level measures, we conducted a sensitivity analysis using an alternative summary metric commonly adopted in prior studies [63,93], namely the L2-norm of the component vectors. Network-level coupling and decoupling were re-computed in the main dataset (126-ROI parcellation) using the L2-norm to quantify component magnitude. Because the L2-norm is sign-invariant, we applied a constant shift to the coupled/decoupled component time series to ensure non-negativity prior to computing the norm.

We focused on the ECN, DMN, and SN—three large-scale brain systems consistently implicated in age-related functional decline [30–34]. In the 17-network parcellation defined by Yeo and colleagues [83], each of these large-scale systems—ECN, DMN, and SN—is subdivided into finer-grained subnetworks that reflect spatially and functionally distinct components. The ECN comprises three subnetworks: ECN-A, primarily involving dorsolateral prefrontal and inferior parietal regions; ECN-B, which includes lateral prefrontal and posterior middle temporal areas; and ECN-C, a more medial and frontal subnetwork that is close to default-mode regions. The DMN includes DMN-A, centered in medial prefrontal and posterior cingulate cortex; DMN-B, which extends into lateral temporal cortex; and DMN-C, a ventromedial prefrontal subsystem. The SN is subdivided into SN-A and SN-B. SN-A primarily involves the anterior insula and dorsal anterior cingulate cortex, while SN-B encompasses regions such as the frontal operculum, temporal-parietal junction, and adjacent lateral prefrontal cortex, and is more closely associated with stimulus-driven attentional reorienting, environmental monitoring, and bottom-up attention capture. To control for multiple comparisons, we restricted the analysis to 3 subnetworks in ECN, 3 in DMN, and 2 in SN, with coupling and decoupling computed for each, resulting in 16 total comparisons.

As a control analysis to contextualize the specificity of effects observed in higher-order cognitive networks, we extended the longitudinal analyses (95% variance) to include the somatomotor and visual networks in addition to the ECN, DMN, and SN subnetworks.

### Estimation of aging-related changes in structure–function integration in older adults

To investigate the longitudinal changes in structure–function coupling and decoupling, we modeled the effects of time on network-level structure–function coupling and decoupling in the main and validation datasets separately using linear mixed effect models, following our previous work [29,42]:


$$Y_{ij}=\;\beta_{\text{0}\text{0}}+\beta_{\text{0}\text{1}}\left({\text{Gender}}_{j}\right)+\beta_{\text{0}\text{2}}\left({\text{Education}}_{j}\right)+\beta_{\text{0}\text{3}}\left({\text{Age}}_{j}\right)+\beta_{\text{1}\text{0}}\left({\text{Time}}_{ij}\right)+\beta_{\text{1}\text{1}}\left({\text{Age}}_{j}*{\text{Time}}_{ij}\right)+\mu_{\text{0}j}+\mu_{\text{1}j}\left({\text{Time}}_{ij}\right)+r_{ij},$$ (5)

where *Y* represented structure–function coupling (or decoupling) from a given network for each participant at each visit; Gender was a binary variable; Age and Education were grand-mean centered baseline age and years of education; Time represented time interval since the first (baseline) session; *β* terms represented fixed effects and μ terms represented random effects for each participant; Subscripts *i* and *j* represented visit and participant, respectively. The fixed-effect coefficient for Time was interpreted as an estimate of the longitudinal (within-subject) effect on structure–function coupling or decoupling. Bonferroni correction was applied across 16 comparisons (8 subnetworks × 2 metrics). In addition, we also applied FDR correction across subnetwork-level tests to provide a complementary, less conservative control of multiple comparisons.

To provide an intuitive summary of the magnitude of longitudinal effects, we additionally quantified the variance explained by the time-related fixed effects using a marginal R^2^ (R^2^m) framework for mixed-effects models [94]. Specifically, we fit a reduced model by removing the time and baseline age × time fixed-effect terms from Equation (5), while keeping the same random-effects structure. We then computed the incremental explained variance as ΔR^2^m = R^2^m(full) − R^2^m(reduced), which represents the additional variance explained by the time-related fixed effects. In parallel, we performed likelihood ratio tests comparing the full and reduced models. To facilitate interpretation, ΔR^2^m values were used as effect-size summaries of time-related longitudinal change and were reported alongside *β* coefficients.

To provide additional insights, we also conducted an exploratory ROI-level analysis of structure–function coupling/decoupling within the ECN, DMN, and SN subnetworks. For each ROI, longitudinal effects were tested using linear mixed-effects models consistent with the main analyses, and ROI-level p-values were corrected for multiple comparisons using Bonferroni correction across ROIs within these networks.

To provide background for interpreting structure–function integration results, we additionally computed mean intrinsic BOLD activity within each ECN, DMN, and SN subnetwork and tested longitudinal effects using the same linear mixed-effects models and related the longitudinal changes in intrinsic subnetwork BOLD activity to corresponding changes in cognitive flexibility.

### Association of structure–function integration with cognitive flexibility

We next examined if longitudinal changes in structure–function coupling and decoupling were associated with corresponding changes in cognitive flexibility. We restricted our analysis to subnetworks that showed significant time effects in the previous longitudinal models. Following previous work [29], we estimated individual longitudinal change rates (*B*_1*j*_) of both structure–function coupling (or decoupling) and cognitive flexibility for each participant using a simple regression model:


$$\overset{\prime }{\underset{ij}{Y}}=\;B_{\text{0}j}+B_{\text{1}j}\left({\text{Time}}_{ij}\right)+r_{ij},$$ (6)

where $\overset{\prime }{\underset{ij}{Y}}\;$ represents the predicted values of structure–function coupling, decoupling, or cognitive flexibility (dTMT scores in the main dataset and dCTT scores in the validation dataset), derived from the linear mixed-effects model described in Equation (5). The slope term $B_{\text{1}j}$ reflects each participant’s rate of change over time.

We then tested whether longitudinal changes in structure–function coupling or decoupling were associated with longitudinal change in cognitive flexibility. This effect was modeled as:


$$B_{\text{1}j.dTMT}=\;b_{\text{0}}+b_{\text{1}}\left(B_{\text{1}j.\text{Coupling}}\right),$$ (7)

$$B_{\text{1}j.dTMT}=\;b_{\text{0}}+b_{\text{1}}\left(B_{\text{1}j.\text{Decoupling}}\right).$$ (8)

All statistical analyses were carried out in MATLAB 2015a (The MathWorks).

## Results

### Network-level structure–function integration showed differential longitudinal changes in older adults

**Fig 2:**
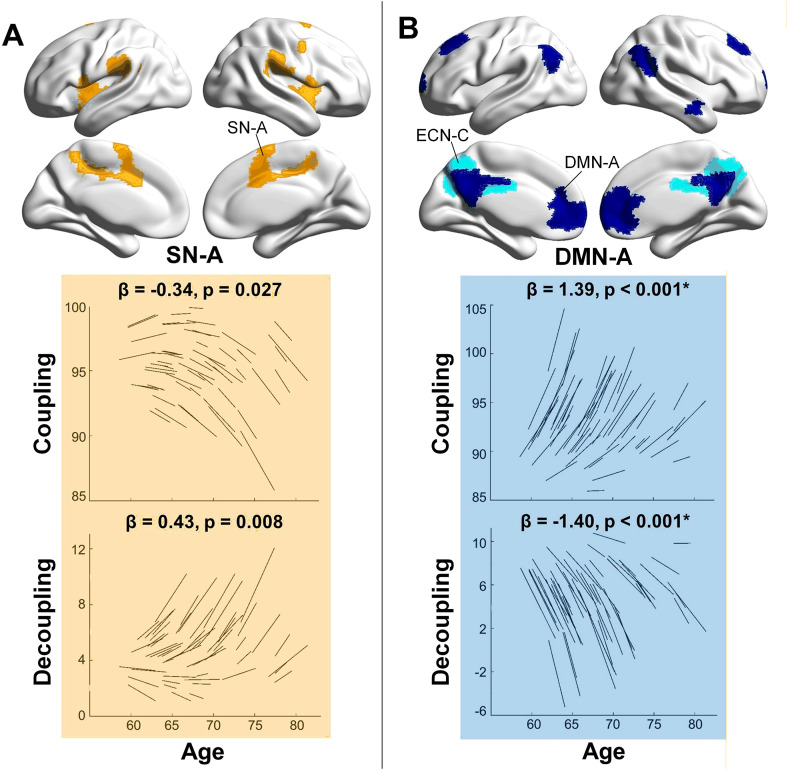
Differential changes in structure–function coupling and decoupling with aging in the main dataset. Longitudinal effects were estimated using linear mixed-effects models, with fixed coefficients (*β*) representing the effect of time interval from the first scan. Spaghetti plots display individual trajectories of structure–function coupling and decoupling over time, adjusted for baseline age, gender, and education. Coupling and decoupling measures were computed using *K* values explaining 95% of variance, shown here for illustration; similar results were observed with 80% and 90% variance thresholds. Asterisks (*) indicate effects that survived Bonferroni correction for multiple comparisons (*α* = 0.05/16 ≈ 0.003). Brain network visualizations were created using BrainNet Viewer (http://www.nitrc.org/projects/bnv). The underlying numerical data for this figure are provided in Supporting information (S1 Data).

In the main dataset, longitudinal changes in structure–function integration were observed within the SN, ECN, and DMN, particularly in the SN-A, DMN-A, and ECN-C subnetworks (Fig 2 and Table A in S1 Appendix). Consistent with our hypothesis, SN-A exhibited a significant decrease in coupling (*p* = 0.027) and a concurrent increase in decoupling (*p* = 0.008) over time, suggesting that functional signals became progressively less constrained by the underlying structural network with aging.

In contrast, DMN-A and ECN-C displayed an opposite pattern: coupling increased (*p* < 0.001 for DMN-A, *p* = 0.018 for ECN-C), while decoupling decreased (*p* < 0.001 for DMN-A, *p* = 0.009 for ECN-C) over time. Notably, ECN-C is anatomically proximal to regions typically associated with the DMN (Fig 2B), making it unsurprising that ECN-C exhibited similar directional changes as DMN-A. These findings may reflect a divergent trajectory of aging-related changes in structure–function integration between task-negative networks (e.g., DMN-A and ECN-C) and task-positive networks (e.g., SN-A).

Of these effects, only DMN-A survived Bonferroni correction for multiple comparisons across coupling and decoupling metrics in all subnetworks (adjusted *α* = 0.05/16 ≈ 0.003). Nonetheless, all observed trends were robust across different values of *K* used in the graph filtering procedure (Table A in S1 Appendix). In addition to Bonferroni-corrected *p*-values, uncorrected and FDR-adjusted *p*-values are reported for subnetwork analyses (see Tables A and B in S1 Appendix).

For structure–function coupling, the additional variance explained by the time-related fixed effects (ΔR^2^m) was 0.025 for SN-A, 0.127 for DMN-A, and 0.032 for ECN-C. Likelihood ratio tests comparing the full and reduced models yielded p-values of 0.025, <0.001, and 0.061, respectively. For structure–function decoupling, the corresponding ΔR^2^m values were 0.052 for SN-A, 0.163 for DMN-A, and 0.049 for ECN-C, with model comparison *p*-values of 0.022, <0.001, and 0.033, respectively.

**Fig 3:**
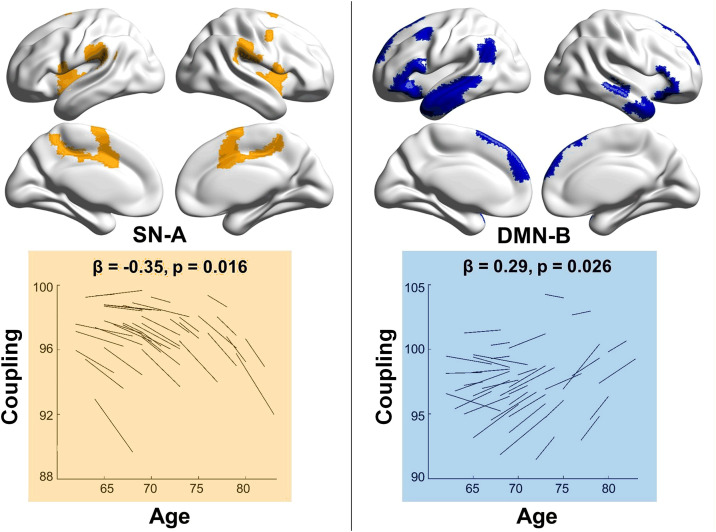
Differential changes in structure–function coupling and decoupling with aging in the validation dataset. Longitudinal effects were estimated using linear mixed-effects models, with fixed coefficients (*β*) representing the effect of time interval from the first scan. Spaghetti plots display individual trajectories of structure–function coupling and decoupling over time, adjusted for baseline age, gender, and education. Coupling and decoupling measures were computed using *K* values explaining 95% of the observed variance, shown here for illustration; similar results were observed with 80% and 90% variance thresholds. Asterisks (*) indicate effects that remained statistically significant after Bonferroni correction for multiple comparisons (*α* = 0.05/16 ≈ 0.003). Brain network visualizations were created using BrainNet Viewer (http://www.nitrc.org/projects/bnv). The underlying numerical data for this figure are provided in Supporting information (S2 Data).

These findings were partially replicated in the validation dataset. Specifically, SN-A showed a significant decline in coupling over time (*p* = 0.016), and along with a trend toward increased decoupling (*p* = 0.072). Additionally, DMN-B exhibited a significant increase in coupling over time (*p* = 0.026) (Fig 3; Table B in S1 Appendix).

Exploratory ROI-level analyses in main dataset revealed patterns broadly consistent with the network-level findings, with the strongest aging-related coupling/decoupling changes observed in ROIs within the SN-A and DMN-A subnetworks (e.g., SalVentAttnA_FrMed and DefaultA_PFCm; Fig C in S1 Appendix).

In a control analysis extending the subnetwork-level longitudinal models in main dataset to include somatomotor and visual networks (in addition to the ECN, DMN, and SN subnetworks), we found that after applying FDR and Bonferroni corrections, the sensorimotor systems did not exhibit significant longitudinal changes in structure–function coupling or decoupling, whereas SN-A, DMN-A, and ECN-C remained significant after correction, indicating robust longitudinal effects. These control analyses provide additional context supporting the relative specificity of the strongest coupling/decoupling alterations in higher-order cognitive and salience/attention-related systems (Table E in S1 Appendix).

In a control analysis examining longitudinal changes in mean SC and intrinsic BOLD activity within ECN, DMN, and SN subnetworks (Table F in S1 Appendix), only intrinsic BOLD activity in DMN-B and SN-A showed significant longitudinal change, with opposite directions (DMN-B increased, whereas SN-A decreased). When relating longitudinal changes in intrinsic BOLD activity in DMN-B and SN-A to corresponding changes in cognitive flexibility, no significant associations were observed (*p* = 0.058 and *p* = 0.18, respectively). Compared with these modest component-level changes, structure–function integration indices showed stronger longitudinal effects, suggesting higher sensitivity to aging-related change in this dataset.

Sensitivity analyses without GSR yielded longitudinal time effects that were virtually identical to the primary results (Table D in S1 Appendix), suggesting that our conclusions are robust to the use of GSR.

### Longitudinal changes in cognitive flexibility were associated with structure–function coupling and decoupling changes in the salience network

**Fig 4:**
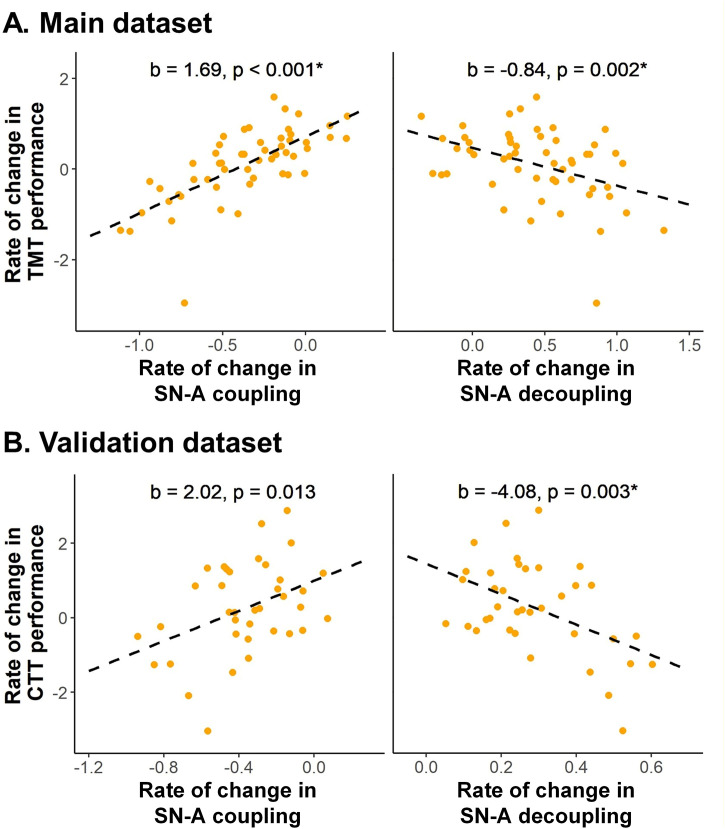
Cognitive flexibility decline over time is associated with changes in salience network structure–function coupling and decoupling in matin and validation datasets. Associations between longitudinal change rates in cognitive flexibility, measured by the Trail Making Test (TMT) in the main dataset (panel **A**) and the Color Trails Test (CTT) in the validation dataset (panel **B**), and change rates in structure–function integration are shown. Scatter plots illustrate the relationship between re-estimated individual slopes (*β*_1*j*_) in TMT (or CTT) performance and coupling/decoupling metrics, adjusted for baseline age, gender, and education. Coupling and decoupling measures were derived using *K* values explaining 95% of the variance; similar results were observed with 80% and 90% thresholds. Asterisks (*) indicate associations that remained statistically significant after Bonferroni correction for multiple comparisons (*α* = 0.05/7 ≈ 0.007). The underlying numerical data for this figure are provided in Supporting information (S3 Data).

Longitudinal changes in cognitive flexibility were significantly associated with longitudinal alterations in structure–function coupling and decoupling within SN-A in both datasets (Fig 4). In the main dataset, a slower rate of decline in cognitive flexibility (as measured by dTMT) was associated with a smaller decrease in SN-A coupling and a smaller increase in SN-A decoupling over time (*b* = 1.69, *p* < 0.001 and *b* = −0.84, *p* = 0.002, respectively). These findings are consistent with previous work in young adults showing that reduced decoupling is associated with lower switch cost during cognitive flexibility tasks [62].

These associations were robust across different values of *K*, and they remained statistically significant after multiple comparisons correction (Table C in S1 Appendix). Importantly, these findings were replicated in the validation dataset, where a slower decline in cognitive flexibility (as indexed by dCTT scores) was associated with a slower decrease in SN-A coupling and a slower increase in SN-A decoupling (*b* = 2.02, *p* = 0.013 and *b* = −4.08, *p* = 0.003, respectively).

Sensitivity analyses using the L2-norm summarization approach yielded longitudinal effects and brain–behavior associations for structure–function coupling that were largely consistent with the main results (Fig B in S1 Appendix).

When replicating the analyses in the main dataset using an alternative parcellation comprising 416 ROIs, the overall findings remained largely consistent with the primary results (Fig A in S1 Appendix). Specifically, the SN-A subnetwork exhibited decreased structure–function coupling and increased decoupling over time, and these longitudinal changes were significantly associated with cognitive flexibility decline as measured by the TMT. In contrast, the DMN-A subnetwork showed the opposite pattern, with increased coupling and decreased decoupling over time. However, ECN-C did not show significant longitudinal effects under the alternative parcellation (*β* ≈ 0, *p* > 0.80). This discrepancy may reflect reduced robustness of the ECN-C effect across parcellations, potentially due to differences in how this relatively small subnetwork is represented (e.g., altered spatial boundaries), which may increase measurement noise and reduce sensitivity to detect subtle longitudinal changes.

## Discussion

Age-related alterations in FC and SC have been widely reported, including reduced SC integrity/efficiency and reduced functional segregation across large-scale systems. Our graph-spectral coupling/decoupling metrics are not intended to replicate these established findings; rather, they provide complementary information by quantifying how the structural connectome constrains intrinsic functional dynamics. In this way, coupling/decoupling indices may offer an additional and potentially more sensitive perspective on aging-related reorganization beyond conventional FC or SC measures alone.

Using two independent longitudinal datasets, we investigated aging-related changes in intrinsic structure–function integration within the subnetworks of three core large-scale cognitive networks, the ECN, DMN, and SN, and examined how these changes relate to longitudinal decline in cognitive flexibility. Overall, we found that both structure–function coupling and decoupling changed over time in normal aging. Notably, these changes followed opposing trajectories in task-positive versus task-negative networks: SN-A exhibited decreasing coupling and increasing decoupling, whereas DMN-A, subnetwork with strong associations to task-negative processing, showed increasing coupling and decreasing decoupling. Crucially, a smaller decline in SN-A coupling and a smaller increase in SN-A decoupling were associated with a slower decline in cognitive flexibility, suggesting that preservation of normative structure–function integration patterns within the SN-A may be critical for maintaining cognitive flexibility in older adults. These findings provide novel insights into how aging alters the degree to which functional brain activity is constrained by SC and demonstrate that such changes are meaningfully linked to aging-related cognitive decline.

### Biological interpretation of structure–function coupling and decoupling in aging

Within the graph-spectral framework, structure–function coupling (alignment) and decoupling (liberality) can be interpreted in relation to low-dimensional bases of brain organization supported by anatomical connectivity. Structural eigenmodes provide spatial bases constrained by the white-matter connectome, such that low-frequency (smooth) modes represent spatially extended, anatomically supported patterns of activity, whereas high-frequency (rough) modes capture more spatially complex patterns that deviate from structural constraints. Increased coupling therefore reflects a shift of intrinsic functional dynamics toward structurally supported low-frequency modes, while reduced decoupling indicates a diminished contribution from higher-frequency, structurally liberal modes, suggesting reduced spatial complexity and flexibility of intrinsic dynamics.

This interpretation is consistent with recent work demonstrating that structural degeneration is associated with reduced amplitudes of intrinsic functional gradients and altered inter-gradient relationships, a pattern interpreted as a form of low-dimensional “network collapse” characterized by increasing dominance of anatomically constrained activity patterns [95]. In this context, the aging-related increase in coupling and decrease in decoupling observed in DMN-A—robust across the main analysis, sensitivity analyses using a finer parcellation resolution, and ROI-level modeling—may reflect a shift toward structurally dominated intrinsic dynamics in higher-order associative systems, which rely on distributed integration and flexible reconfiguration to support executive function.

### Maintenance of normative salience network structure–function integration in aging supports cognitive flexibility

Among the observed aging-related changes in structure–function coupling and decoupling, the longitudinal time effects in SN-A were consistent with our hypothesis, showing a decline in coupling and an increase in decoupling with age. While prior studies have reported mixed findings regarding age-related functional changes in the SN [96–100], there is more consistent evidence of gray matter loss and white matter tract deterioration in SN regions with aging [98,101–103]. This structural degradation may force greater reliance on indirect anatomical pathways for signal transmission [27,97], potentially reflecting a compensatory shift in network dynamics aimed at preserving SN functionality. Such a shift would manifest as decreased coupling and increased decoupling between functional activity and the underlying structural architecture.

Although structural atrophy and white matter decline are common across multiple brain systems in aging, the SN may be particularly sensitive to disruptions in structure–function integration due to its pivotal role in network switching and brain state transitions [104–106]. Notably, the SN is composed of distinct subnetworks with different functional profiles. SN-A primarily includes regions such as the anterior insula and dorsal anterior cingulate cortex, which are involved in salience detection, interoceptive awareness, and initiating dynamic switching between the DMN and ECN, even during a task-free resting state [107,108]. In contrast, SN-B includes areas such as the frontal operculum and temporoparietal junction, and is more closely associated with stimulus-driven attentional reorienting, bottom-up attention capture, and environmental monitoring. This functional dissociation allows the broader SN to flexibly coordinate internal and external attention based on changing task demands. Our finding of significant aging-related changes in structure–function integration in SN-A, but not SN-B, may therefore reflect differential vulnerability of these SN subsystems to aging-related structural deterioration [31]. While ROI-level findings provide finer-grained localization, we interpret them as exploratory given the multiple-comparison burden and potential parcel-level noise; nevertheless, their consistency with network-level results supports the robustness of our main conclusions.

Importantly, individuals with greater reductions in SN-A coupling and greater increases in decoupling also exhibited steeper declines in cognitive flexibility. These associations align with prior work [62], which found that reduced global liberality (decoupling) was linked to better cognitive switching performance in younger adults. Taken together, these findings support the idea that there is an optimal level of structure–function integration, and that aberrant integration, particularly in the SN, may impair cognitive flexibility. Given the SN’s critical role in task-set reconfiguration and network switching, optimal structure–function integration within this network is likely essential for efficiently adapting to task demands. This possibility is further supported by studies showing that SN connectivity correlates with individual differences in cognitive flexibility [109,110], while loss of SN connectivity impairs such abilities [111,112].

### Differential structure–function integration changes in task-positive and task-negative networks in aging

In contrast, longitudinal changes in ECN-C and DMN-A were opposite in direction to those observed in SN-A. Both subnetworks exhibited increased coupling and decreased decoupling with aging. As ECN-C includes regions anatomically adjacent to the precuneus and posterior cingulate cortex, key nodes of the DMN [34,113], it is plausible that ECN-C shows convergence toward DMN-like organization. Notably, however, this ECN-C effect was smaller in magnitude and was attenuated in sensitivity analyses using a finer parcellation resolution and in ROI-level modeling, suggesting it may be less robust than the DMN-A and SN-A findings. Prior work has suggested that the long-range functional connections of the DMN are disproportionately impacted by aging, leading to increased reliance on short-range connectivity [114,115]. Given that diffusion MRI and tractography may be limited in estimation of longer-range neuroanatomical projections [116], short-range structural connections may contribute more strongly to the SC matrices used in our analysis. Thus, increased coupling and reduced decoupling in the DMN may reflect a greater functional dependency on short-range, structurally mediated connections in aging.

However, the DMN is known to include hub regions that are highly integrated with other networks throughout the brain [117,118]. As such, a shift toward short-range connectivity may signify a less efficient utilization of neural resources, undermining the DMN’s ability to coordinate distributed activity and perform complex internal tasks [119,120]. This interpretation is consistent with prior work [27], suggesting that hub-like regions are more susceptible to aging-related declines in communication efficiency. This inefficiency may be further magnified during resting-state conditions, where the DMN is highly active and contributes to maintaining optimal transitions between brain states [121]. As such, the higher coupling and lower decoupling observed in aging DMN networks may underlie reduced functional specialization, with the DMN potentially relying on compensatory recruitment of other networks to maintain its functions [27,29,97]. While we speculated that this pattern may involve greater reliance on short-range connectivity and reduced network efficiency, we emphasize that this interpretation is indirect. Rather, our findings suggest that aging may bias intrinsic functional dynamics in DMN toward more locally coherent, structurally constrained modes, consistent with reduced flexibility of large-scale network organization. Future work integrating graph-spectral measures with explicit metrics of connection length, efficiency, and functional gradients will be important to directly test these mechanistic hypotheses.

Our finding that task-positive SN-A showed the opposite trajectory from DMN-A supports the notion that task-positive and task-negative networks exhibit distinct structural–functional adaptations in aging. We speculate that task-positive networks may require greater functional flexibility to sustain performance in the face of structural decline, thus showing increased decoupling and reduced coupling over time. These compensatory effects may become increasingly important in older adults and manifest as greater divergence between functional signals and structural constraints. By contrast, task-negative networks such as the DMN may shift toward more rigid, structure-driven dynamics, potentially reflecting reduced efficiency in network-level coordination. Additionally, neurovascular differences between task-positive and task-negative networks [122] may further contribute to these divergent trajectories, though future work is needed to explore this possibility in detail.

Our control analyses further indicate that aging-related changes in structure–function integration are network-dependent. Higher-order attention/salience-related systems (e.g., SN and DAN) showed a pattern of reduced coupling and increased decoupling, whereas primary sensorimotor networks showed a different profile (e.g., reduced decoupling with largely stable coupling), consistent with their stronger structural constraints and lower position along the cortical functional hierarchy.

### Limitations and future directions

Several limitations should be acknowledged in the current study. First, although we identified aging-related changes in structure–function integration, most of these effects did not survive correction for multiple comparisons. To mitigate this limitation, we reported only results that were consistent across different *K* values and partially replicated in an independent dataset, which strengthens the robustness and interpretability of our findings. Second, our sample primarily included older adults, which may limit the generalizability of our findings across the full adult life span. Future studies could incorporate a broader age range to better characterize the trajectories of structure–function integration across the life span and to capture potential critical inflection points in aging-related neural reorganization. Third, vascular health and reactivity may influence the hemodynamic properties of the BOLD signal, particularly in older adults, where neurovascular coupling may be altered [123,124]. Although we attempted to control for this confound by including only participants without a history of major vascular events (main dataset) or cerebrovascular disease (validation dataset), residual vascular effects may still influence estimates of structure–function integration. Future work could incorporate quantitative assessments of vascular health, such as arterial stiffness, cerebral blood flow, or cerebrovascular reactivity, to better isolate neural-specific changes from vascular confounds. Although our study focused on normal aging, the structure–function integration framework may also be valuable for understanding pathological aging and neurodegenerative diseases. Future research could extend this approach by incorporating Alzheimer’s disease–related pathologies, such as amyloid burden. In addition, our analyses focused on static, network-level measures of structure–function integration during resting-state fMRI. Future research could extend this work by applying the framework to dynamic or task-based fMRI, particularly in tasks involving cognitive switching. Such investigations could offer a more granular, time-resolved understanding of how anatomical constraints shape neural activity patterns and behavior on a moment-to-moment basis.

## Conclusions

In conclusion, we found that network-level structure–function integration undergoes significant changes with aging, with task-positive SN-A showing decreased coupling and increased decoupling, while task-negative DMN regions exhibited the opposite trend. These divergent trajectories suggest that the degree to which structural networks constrain functional signals in normal aging may be shaped by the functional role of each network, potentially reflecting differences in intrinsic activity, organizational complexity, or efficiency across task-positive and task-negative systems. Importantly, we also observed that age-related changes in SN-A coupling and decoupling were associated with longitudinal changes in cognitive flexibility, underscoring the relevance of structure–function integration for supporting executive function in aging. Together, our findings suggest that aging-related changes in structure–function integration may reflect both network-specific vulnerabilities of the structural connectome and adaptive responses to preserve cognitive performance. These results highlight the value of multimodal analyses for understanding the neurobiological underpinnings of cognitive aging and point to structure–function integration as a promising marker for cognitive resilience in later life.

## Supporting information

S1 Appendix **Table A.** Age and time effects for structure–function coupling and decoupling of all networks of interest in main dataset. **Table B.** Age and time effects for structure–function coupling and decoupling of all networks of interest in validation dataset. **Table C.** Association between changes in structure–function coupling/decoupling and cognitive flexibility scores in main and validation datasets. **Table D.** Sensitivity analysis without global signal regression (GSR): Age and time effects for structure–function coupling and decoupling in main dataset. **Table E.** Time effects for structure–function coupling and decoupling in main dataset (including sensorimotor networks). **Table F.** Age and time effects for BOLD activity in main dataset. **Fig A**. Longitudinal changes in structure–function coupling and decoupling with aging and their associations with cognitive flexibility decline in the main dataset (validation analysis using a 416-ROI parcellation). **Fig B.** Longitudinal changes in structure–function integration with aging and the associations with cognitive flexibility decline in the main dataset (sensitivity analysis using the L2-norm to quantify component magnitude). **Fig C.** ROI-level longitudinal changes in structure–function integration in the main dataset (exploratory analysis within the DMN, ECN, and SN).
(DOCX)

S1 Data The underlying numerical data for Fig 2. (XLSX)

S2 Data The underlying numerical data for Fig 3. (XLSX)

S3 Data The underlying numerical data for Fig 4. (XLSX)

## Abbreviations

CSF: cerebrospinal fluid
CTT: Color Trails Test
DMN: default mode network
dMRI: diffusion-weighted MRI
ECN: executive control network
FC: functional connectivity
GFT: graph Fourier transform
GSR: global signal regression
MPRAGE: magnetization prepared rapid acquisition gradient echo
ROI: regions of interest
SC: structural connectivity
SN: salience network
TMT: Trail Making Test

## References

[1] Diamond A. Executive functions. Annu Rev Psychol. 2013;64:135–68. doi: 10.1146/annurev-psych-113011-143750 23020641 PMC4084861

[2] Jurado MB, Rosselli M. The elusive nature of executive functions: a review of our current understanding. Neuropsychol Rev. 2007;17(3):213–33. doi: 10.1007/s11065-007-9040-z 17786559

[3] Miyake A, Friedman NP, Emerson MJ, Witzki AH, Howerter A, Wager TD. The unity and diversity of executive functions and their contributions to complex “Frontal Lobe” tasks: a latent variable analysis. Cogn Psychol. 2000;41(1):49–100. doi: 10.1006/cogp.1999.0734 10945922

[4] Dajani DR, Uddin LQ. Demystifying cognitive flexibility: implications for clinical and developmental neuroscience. Trends Neurosci. 2015;38(9):571–8. doi: 10.1016/j.tins.2015.07.003 26343956 PMC5414037

[5] Uddin LQ. Cognitive and behavioural flexibility: neural mechanisms and clinical considerations. Nat Rev Neurosci. 2021;22(3):167–79. doi: 10.1038/s41583-021-00428-w 33536614 PMC7856857

[6] Uddin LQ. Cognitive and behavioural flexibility: neural mechanisms and clinical considerations. Nat Rev Neurosci. 2021;22(3):167–79. doi: 10.1038/s41583-021-00428-w 33536614 PMC7856857

[7] Schwarze SA, Fandakova Y, Lindenberger U. Cognitive flexibility across the lifespan: developmental differences in the neural basis of sustained and transient control processes during task switching. Curr Opin Behav Sci. 2024;58:101395. doi: 10.1016/j.cobeha.2024.101395

[8] El-Sayed MM, Hawash MM, Khedr MA, Hafez SA, Salem E-SAE-H, Essa SA, et al. Cognitive flexibility’s role in shaping self-perception of aging, body appreciation, and self-efficacy among community-dwelling older women. BMC Nurs. 2024;23(1):220. doi: 10.1186/s12912-024-01874-4 38561732 PMC10983730

[9] Amelchenko EM, Bezriadnov DV, Chekhov OA, Anokhin KV, Lazutkin AA, Enikolopov G. Age-related decline in cognitive flexibility is associated with the levels of hippocampal neurogenesis. Front Neurosci. 2023;17:1232670. doi: 10.3389/fnins.2023.1232670 37645372 PMC10461065

[10] Cox SR, Ritchie SJ, Tucker-Drob EM, Liewald DC, Hagenaars SP, Davies G, et al. Ageing and brain white matter structure in 3,513 UK Biobank participants. Nat Commun. 2016;7:13629. doi: 10.1038/ncomms13629 27976682 PMC5172385

[11] Hong Z, Ng KK, Sim SKY, Ngeow MY, Zheng H, Lo JC, et al. Differential age-dependent associations of gray matter volume and white matter integrity with processing speed in healthy older adults. Neuroimage. 2015;123:42–50. doi: 10.1016/j.neuroimage.2015.08.034 26302672

[12] Salat DH, Tuch DS, Greve DN, van der Kouwe AJW, Hevelone ND, Zaleta AK, et al. Age-related alterations in white matter microstructure measured by diffusion tensor imaging. Neurobiol Aging. 2005;26(8):1215–27. doi: 10.1016/j.neurobiolaging.2004.09.017 15917106

[13] Good CD, Johnsrude IS, Ashburner J, Henson RN, Friston KJ, Frackowiak RS. A voxel-based morphometric study of ageing in 465 normal adult human brains. Neuroimage. 2001;14(1 Pt 1):21–36. doi: 10.1006/nimg.2001.0786 11525331

[14] Leong RLF, Lo JC, Sim SKY, Zheng H, Tandi J, Zhou J, et al. Longitudinal brain structure and cognitive changes over 8 years in an East Asian cohort. Neuroimage. 2017;147:852–60. doi: 10.1016/j.neuroimage.2016.10.016 27742600

[15] Smith CD, Chebrolu H, Wekstein DR, Schmitt FA, Markesbery WR. Age and gender effects on human brain anatomy: a voxel-based morphometric study in healthy elderly. Neurobiol Aging. 2007;28(7):1075–87. doi: 10.1016/j.neurobiolaging.2006.05.018 16774798

[16] Sowell ER, Peterson BS, Thompson PM, Welcome SE, Henkenius AL, Toga AW. Mapping cortical change across the human life span. Nat Neurosci. 2003;6(3):309–15. doi: 10.1038/nn1008 12548289

[17] Lemaitre H, Goldman AL, Sambataro F, Verchinski BA, Meyer-Lindenberg A, Weinberger DR, et al. Normal age-related brain morphometric changes: nonuniformity across cortical thickness, surface area and gray matter volume?. Neurobiol Aging. 2012;33(3):617.e1-9. doi: 10.1016/j.neurobiolaging.2010.07.013 20739099 PMC3026893

[18] Salat DH, Buckner RL, Snyder AZ, Greve DN, Desikan RSR, Busa E, et al. Thinning of the cerebral cortex in aging. Cereb Cortex. 2004;14(7):721–30. doi: 10.1093/cercor/bhh032 15054051

[19] Shaw ME, Sachdev PS, Anstey KJ, Cherbuin N. Age-related cortical thinning in cognitively healthy individuals in their 60s: the PATH Through Life study. Neurobiol Aging. 2016;39:202–9. doi: 10.1016/j.neurobiolaging.2015.12.009 26923417

[20] Cabeza R, Daselaar SM, Dolcos F, Prince SE, Budde M, Nyberg L. Task-independent and task-specific age effects on brain activity during working memory, visual attention and episodic retrieval. Cereb Cortex. 2004;14(4):364–75. doi: 10.1093/cercor/bhg133 15028641

[21] Grady CL, Maisog JM, Horwitz B, Ungerleider LG, Mentis MJ, Salerno JA, et al. Age-related changes in cortical blood flow activation during visual processing of faces and location. J Neurosci. 1994;14(3 Pt 2):1450–62. doi: 10.1523/JNEUROSCI.14-03-01450.1994 8126548 PMC6577560

[22] Li H-J, Hou X-H, Liu H-H, Yue C-L, Lu G-M, Zuo X-N. Putting age-related task activation into large-scale brain networks: a meta-analysis of 114 fMRI studies on healthy aging. Neurosci Biobehav Rev. 2015;57:156–74. doi: 10.1016/j.neubiorev.2015.08.013 26318367

[23] Madden DJ, Turkington TG, Coleman RE, Provenzale JM, DeGrado TR, Hoffman JM. Adult age differences in regional cerebral blood flow during visual world identification: evidence from H215O PET. Neuroimage. 1996;3(2):127–42. doi: 10.1006/nimg.1996.0015 9345484

[24] Spreng RN, Wojtowicz M, Grady CL. Reliable differences in brain activity between young and old adults: a quantitative meta-analysis across multiple cognitive domains. Neurosci Biobehav Rev. 2010;34(8):1178–94. doi: 10.1016/j.neubiorev.2010.01.009 20109489

[25] Damoiseaux JS, Beckmann CF, Arigita EJS, Barkhof F, Scheltens P, Stam CJ, et al. Reduced resting-state brain activity in the “default network” in normal aging. Cereb Cortex. 2008;18(8):1856–64. doi: 10.1093/cercor/bhm207 18063564

[26] Andrews-Hanna JR, Snyder AZ, Vincent JL, Lustig C, Head D, Raichle ME, et al. Disruption of large-scale brain systems in advanced aging. Neuron. 2007;56(5):924–35. doi: 10.1016/j.neuron.2007.10.038 18054866 PMC2709284

[27] Betzel RF, Byrge L, He Y, Goñi J, Zuo X-N, Sporns O. Changes in structural and functional connectivity among resting-state networks across the human lifespan. Neuroimage. 2014;102 Pt 2:345–57. doi: 10.1016/j.neuroimage.2014.07.067 25109530

[28] Geerligs L, Maurits NM, Renken RJ, Lorist MM. Reduced specificity of functional connectivity in the aging brain during task performance. Hum Brain Mapp. 2014;35(1):319–30. doi: 10.1002/hbm.22175 22915491 PMC6869200

[29] Ng KK, Lo JC, Lim JKW, Chee MWL, Zhou J. Reduced functional segregation between the default mode network and the executive control network in healthy older adults: a longitudinal study. Neuroimage. 2016;133:321–30. doi: 10.1016/j.neuroimage.2016.03.029 27001500

[30] Spreng RN, Stevens WD, Viviano JD, Schacter DL. Attenuated anticorrelation between the default and dorsal attention networks with aging: evidence from task and rest. Neurobiol Aging. 2016;45:149–60. doi: 10.1016/j.neurobiolaging.2016.05.020 27459935 PMC5003045

[31] Touroutoglou A, et al. Dissociable effects of aging on salience subnetwork connectivity mediate age-related changes in executive function and affect. Front Aging Neurosci. 2018;10:410. 30618717 10.3389/fnagi.2018.00410 PMC6304391

[32] Turner GR, Spreng RN. Prefrontal engagement and reduced default network suppression co-occur and are dynamically coupled in older adults: the default-executive coupling hypothesis of aging. J Cogn Neurosci. 2015;27(12):2462–76. doi: 10.1162/jocn_a_00869 26351864

[33] Seeley WW, Menon V, Schatzberg AF, Keller J, Glover GH, Kenna H, et al. Dissociable intrinsic connectivity networks for salience processing and executive control. J Neurosci. 2007;27(9):2349–56. doi: 10.1523/JNEUROSCI.5587-06.2007 17329432 PMC2680293

[34] Buckner RL, Andrews-Hanna JR, Schacter DL. The brain’s default network: anatomy, function, and relevance to disease. Ann N Y Acad Sci. 2008;1124:1–38. doi: 10.1196/annals.1440.011 18400922

[35] Menon V, Uddin LQ. Saliency, switching, attention and control: a network model of insula function. Brain Struct Funct. 2010;214(5–6):655–67. doi: 10.1007/s00429-010-0262-0 20512370 PMC2899886

[36] Gong G, Rosa-Neto P, Carbonell F, Chen ZJ, He Y, Evans AC. Age- and gender-related differences in the cortical anatomical network. J Neurosci. 2009;29(50):15684–93. doi: 10.1523/JNEUROSCI.2308-09.2009 20016083 PMC2831804

[37] Wu K, Taki Y, Sato K, Kinomura S, Goto R, Okada K, et al. Age-related changes in topological organization of structural brain networks in healthy individuals. Hum Brain Mapp. 2012;33(3):552–68. doi: 10.1002/hbm.21232 21391279 PMC6870030

[38] Zhao T, Cao M, Niu H, Zuo X-N, Evans A, He Y, et al. Age-related changes in the topological organization of the white matter structural connectome across the human lifespan. Hum Brain Mapp. 2015;36(10):3777–92. doi: 10.1002/hbm.22877 26173024 PMC6869038

[39] Zhu W, Wen W, He Y, Xia A, Anstey KJ, Sachdev P. Changing topological patterns in normal aging using large-scale structural networks. Neurobiol Aging. 2012;33(5):899–913. doi: 10.1016/j.neurobiolaging.2010.06.022 20724031

[40] Fjell AM, Sneve MH, Storsve AB, Grydeland H, Yendiki A, Walhovd KB. Brain events underlying episodic memory changes in aging: a longitudinal investigation of structural and functional connectivity. Cereb Cortex. 2016;26(3):1272–86. doi: 10.1093/cercor/bhv102 25994960 PMC4737610

[41] Zimmermann J, Ritter P, Shen K, Rothmeier S, Schirner M, McIntosh AR. Structural architecture supports functional organization in the human aging brain at a regionwise and network level. Hum Brain Mapp. 2016;37(7):2645–61. doi: 10.1002/hbm.23200 27041212 PMC6867479

[42] Chong JSX, Ng KK, Tandi J, Wang C, Poh J-H, Lo JC, et al. Longitudinal changes in the cerebral cortex functional organization of healthy elderly. J Neurosci. 2019;39(28):5534–50. doi: 10.1523/JNEUROSCI.1451-18.2019 31109962 PMC6616287

[43] O’Sullivan M, Jones DK, Summers PE, Morris RG, Williams SC, Markus HS. Evidence for cortical “disconnection” as a mechanism of age-related cognitive decline. Neurology. 2001;57(4):632–8. doi: 10.1212/wnl.57.4.632 11524471

[44] Chong JSX, Ng KK, Tandi J, Wang C, Poh J-H, Lo JC, et al. Longitudinal changes in the cerebral cortex functional organization of healthy elderly. J Neurosci. 2019;39(28):5534–50. doi: 10.1523/JNEUROSCI.1451-18.2019 31109962 PMC6616287

[45] Deco G, Ponce-Alvarez A, Mantini D, Romani GL, Hagmann P, Corbetta M. Resting-state functional connectivity emerges from structurally and dynamically shaped slow linear fluctuations. J Neurosci. 2013;33(27):11239–52. doi: 10.1523/JNEUROSCI.1091-13.2013 23825427 PMC3718368

[46] Goñi J, van den Heuvel MP, Avena-Koenigsberger A, Velez de Mendizabal N, Betzel RF, Griffa A, et al. Resting-brain functional connectivity predicted by analytic measures of network communication. Proc Natl Acad Sci U S A. 2014;111(2):833–8. doi: 10.1073/pnas.1315529111 24379387 PMC3896172

[47] Hermundstad AM, Bassett DS, Brown KS, Aminoff EM, Clewett D, Freeman S, et al. Structural foundations of resting-state and task-based functional connectivity in the human brain. Proc Natl Acad Sci U S A. 2013;110(15):6169–74. doi: 10.1073/pnas.1219562110 23530246 PMC3625268

[48] Honey CJ, Sporns O, Cammoun L, Gigandet X, Thiran JP, Meuli R, et al. Predicting human resting-state functional connectivity from structural connectivity. Proc Natl Acad Sci U S A. 2009;106(6):2035–40. doi: 10.1073/pnas.0811168106 19188601 PMC2634800

[49] Skudlarski P, Jagannathan K, Calhoun VD, Hampson M, Skudlarska BA, Pearlson G. Measuring brain connectivity: diffusion tensor imaging validates resting state temporal correlations. Neuroimage. 2008;43(3):554–61. doi: 10.1016/j.neuroimage.2008.07.063 18771736 PMC4361080

[50] van den Heuvel MP, Mandl RCW, Kahn RS, Hulshoff Pol HE. Functionally linked resting-state networks reflect the underlying structural connectivity architecture of the human brain. Hum Brain Mapp. 2009;30(10):3127–41. doi: 10.1002/hbm.20737 19235882 PMC6870902

[51] Barttfeld P, Uhrig L, Sitt JD, Sigman M, Jarraya B, Dehaene S. Signature of consciousness in the dynamics of resting-state brain activity. Proc Natl Acad Sci U S A. 2015;112(3):887–92. doi: 10.1073/pnas.1418031112 25561541 PMC4311826

[52] Hahn G, Zamora-López G, Uhrig L, Tagliazucchi E, Laufs H, Mantini D, et al. Signature of consciousness in brain-wide synchronization patterns of monkey and human fMRI signals. Neuroimage. 2021;226:117470. doi: 10.1016/j.neuroimage.2020.117470 33137478

[53] Tagliazucchi E, Crossley N, Bullmore ET, Laufs H. Deep sleep divides the cortex into opposite modes of anatomical-functional coupling. Brain Struct Funct. 2016;221(8):4221–34. doi: 10.1007/s00429-015-1162-0 26650048

[54] Romero-Garcia R, Atienza M, Cantero JL. Predictors of coupling between structural and functional cortical networks in normal aging. Hum Brain Mapp. 2014;35(6):2724–40. doi: 10.1002/hbm.22362 24027166 PMC6869592

[55] Yang Z, Chang C, Xu T, Jiang L, Handwerker DA, Castellanos FX, et al. Connectivity trajectory across lifespan differentiates the precuneus from the default network. Neuroimage. 2014;89:45–56. doi: 10.1016/j.neuroimage.2013.10.039 24287438 PMC3944140

[56] Wang J, Khosrowabadi R, Ng KK, Hong Z, Chong JSX, Wang Y, et al. Alterations in brain network topology and structural-functional connectome coupling relate to cognitive impairment. Front Aging Neurosci. 2018;10:404. doi: 10.3389/fnagi.2018.00404 30618711 PMC6300727

[57] Cocchi L, Harding IH, Lord A, Pantelis C, Yucel M, Zalesky A. Disruption of structure-function coupling in the schizophrenia connectome. Neuroimage Clin. 2014;4:779–87. doi: 10.1016/j.nicl.2014.05.004 24936428 PMC4055899

[58] Koubiyr I, Deloire M, Brochet B, Besson P, Charré-Morin J, Saubusse A, et al. Structural constraints of functional connectivity drive cognitive impairment in the early stages of multiple sclerosis. Mult Scler. 2021;27(4):559–67. doi: 10.1177/1352458520971807 33283582

[59] Wang S, Gan S, Yang X, Li T, Xiong F, Jia X, et al. Decoupling of structural and functional connectivity in hubs and cognitive impairment after mild traumatic brain injury. Brain Connect. 2021;11(9):745–58. doi: 10.1089/brain.2020.0852 33605188

[60] Damoiseaux JS, Greicius MD. Greater than the sum of its parts: a review of studies combining structural connectivity and resting-state functional connectivity. Brain Struct Funct. 2009;213(6):525–33. doi: 10.1007/s00429-009-0208-6 19565262

[61] Suarez LE, Markello RD, Betzel RF, Misic B. Linking structure and function in macroscale brain networks. Trends Cogn Sci. 2020;24(4):302–15. 32160567 10.1016/j.tics.2020.01.008

[62] Medaglia JD, Huang W, Karuza EA, Kelkar A, Thompson-Schill SL, Ribeiro A, et al. Functional alignment with anatomical networks is associated with cognitive flexibility. Nat Hum Behav. 2018;2(2):156–64. doi: 10.1038/s41562-017-0260-9 30498789 PMC6258039

[63] Preti MG, Van De Ville D. Decoupling of brain function from structure reveals regional behavioral specialization in humans. Nat Commun. 2019;10(1):4747. doi: 10.1038/s41467-019-12765-7 31628329 PMC6800438

[64] Lezak MD, Howieson DB, Loring DW, Fischer JS. Neuropsychological assessment. USA: Oxford University Press; 2004.

[65] Ridderinkhof KR, Span MM, van der Molen MW. Perseverative behavior and adaptive control in older adults: performance monitoring, rule induction, and set shifting. Brain Cogn. 2002;49(3):382–401. doi: 10.1006/brcg.2001.1506 12139960

[66] Verhaeghen P, Cerella J. Aging, executive control, and attention: a review of meta-analyses. Neurosci Biobehav Rev. 2002;26(7):849–57. doi: 10.1016/s0149-7634(02)00071-4 12470697

[67] Wasylyshyn C, Verhaeghen P, Sliwinski MJ. Aging and task switching: a meta-analysis. Psychol Aging. 2011;26(1):15–20. doi: 10.1037/a0020912 21261411 PMC4374429

[68] Wecker NS, Kramer JH, Hallam BJ, Delis DC. Mental flexibility: age effects on switching. Neuropsychology. 2005;19(3):345–52. doi: 10.1037/0894-4105.19.3.345 15910120

[69] Gold BT, Powell DK, Xuan L, Jicha GA, Smith CD. Age-related slowing of task switching is associated with decreased integrity of frontoparietal white matter. Neurobiol Aging. 2010;31(3):512–22. doi: 10.1016/j.neurobiolaging.2008.04.005 18495298 PMC2815097

[70] Hakun JG, Zhu Z, Johnson NF, Gold BT. Evidence for reduced efficiency and successful compensation in older adults during task switching. Cortex. 2015;64:352–62. doi: 10.1016/j.cortex.2014.12.006 25614233 PMC4346415

[71] Kunimi M, Kiyama S, Nakai T. Investigation of age-related changes in brain activity during the divalent task-switching paradigm using functional MRI. Neurosci Res. 2016;103:18–26. doi: 10.1016/j.neures.2015.06.011 26193452

[72] Kupis L, Goodman ZT, Kornfeld S, Hoang S, Romero C, Dirks B, et al. Brain dynamics underlying cognitive flexibility across the lifespan. Cereb Cortex. 2021;31(11):5263–74. doi: 10.1093/cercor/bhab156 34145442 PMC8491685

[73] Chee MWL, Chen KHM, Zheng H, Chan KPL, Isaac V, Sim SKY, et al. Cognitive function and brain structure correlations in healthy elderly East Asians. Neuroimage. 2009;46(1):257–69. doi: 10.1016/j.neuroimage.2009.01.036 19457386

[74] Chong JSX, Liu S, Loke YM, Hilal S, Ikram MK, Xu X, et al. Influence of cerebrovascular disease on brain networks in prodromal and clinical Alzheimer’s disease. Brain. 2017;140(11):3012–22. doi: 10.1093/brain/awx224 29053778 PMC5841199

[75] Hilal S, Chai YL, Ikram MK, Elangovan S, Yeow TB, Xin X, et al. Markers of cardiac dysfunction in cognitive impairment and dementia. Medicine (Baltimore). 2015;94(1):e297. doi: 10.1097/MD.0000000000000297 25569645 PMC4602830

[76] Reitan RM, Wolfson D. The Halstead-Reitan neuropsychological test battery: theory and clinical interpretation. Reitan Neuropsychology. 1985;4.

[77] Lo JC, Loh KK, Zheng H, Sim SKY, Chee MWL. Sleep duration and age-related changes in brain structure and cognitive performance. Sleep. 2014;37(7):1171–8. doi: 10.5665/sleep.3832 25061245 PMC4098802

[78] D’Elia L, Satz P, Uchiyama CL, White T. Color trails test. Odessa, FL: PAR; 1996.

[79] Lim MJR, Tan CS, Gyanwali B, Chen C, Hilal S. The effect of intracranial stenosis on cognitive decline in a memory clinic cohort. Eur J Neurol. 2021;28(6):1829–39. doi: 10.1111/ene.14788 33630355

[80] Yeo D, et al. Pilot validation of a customized neuropsychological battery in elderly Singaporeans. Neurol J South East Asia. 1997;2(123).

[81] Jenkinson M et al. Fsl. Neuroimage. 2012;62(2):782–90. 21979382 10.1016/j.neuroimage.2011.09.015

[82] Cox RW. AFNI: software for analysis and visualization of functional magnetic resonance neuroimages. Comput Biomed Res. 1996;29(3):162–73. doi: 10.1006/cbmr.1996.0014 8812068

[83] Yeo BTT, Krienen FM, Sepulcre J, Sabuncu MR, Lashkari D, Hollinshead M, et al. The organization of the human cerebral cortex estimated by intrinsic functional connectivity. J Neurophysiol. 2011;106(3):1125–65. doi: 10.1152/jn.00338.2011 21653723 PMC3174820

[84] Tzourio-Mazoyer N, Landeau B, Papathanassiou D, Crivello F, Etard O, Delcroix N, et al. Automated anatomical labeling of activations in SPM using a macroscopic anatomical parcellation of the MNI MRI single-subject brain. Neuroimage. 2002;15(1):273–89. doi: 10.1006/nimg.2001.0978 11771995

[85] Zuo X-N, Xing X-X. Test-retest reliabilities of resting-state FMRI measurements in human brain functional connectomics: a systems neuroscience perspective. Neurosci Biobehav Rev. 2014;45:100–18. doi: 10.1016/j.neubiorev.2014.05.009 24875392

[86] Zhao T, Duan F, Liao X, Dai Z, Cao M, He Y, et al. Test-retest reliability of white matter structural brain networks: a multiband diffusion MRI study. Front Hum Neurosci. 2015;9:59. doi: 10.3389/fnhum.2015.00059 25741265 PMC4330899

[87] Schaefer A, Kong R, Gordon EM, Laumann TO, Zuo X-N, Holmes AJ, et al. Local-global parcellation of the human cerebral cortex from intrinsic functional connectivity MRI. Cereb Cortex. 2018;28(9):3095–114. doi: 10.1093/cercor/bhx179 28981612 PMC6095216

[88] Tian Y, Margulies DS, Breakspear M, Zalesky A. Topographic organization of the human subcortex unveiled with functional connectivity gradients. Nat Neurosci. 2020;23(11):1421–32. 32989295 10.1038/s41593-020-00711-6

[89] Behrens TEJ, Woolrich MW, Jenkinson M, Johansen-Berg H, Nunes RG, Clare S, et al. Characterization and propagation of uncertainty in diffusion-weighted MR imaging. Magn Reson Med. 2003;50(5):1077–88. doi: 10.1002/mrm.10609 14587019

[90] Behrens TEJ, Berg HJ, Jbabdi S, Rushworth MFS, Woolrich MW. Probabilistic diffusion tractography with multiple fibre orientations: What can we gain?. Neuroimage. 2007;34(1):144–55. doi: 10.1016/j.neuroimage.2006.09.018 17070705 PMC7116582

[91] Cui Z, Zhong S, Xu P, He Y, Gong G. PANDA: a pipeline toolbox for analyzing brain diffusion images. Front Hum Neurosci. 2013;7:42. doi: 10.3389/fnhum.2013.00042 23439846 PMC3578208

[92] Zhang L, Biessels GJ, Hilal S, Chong JSX, Liu S, Shim HY, et al. Cerebral microinfarcts affect brain structural network topology in cognitively impaired patients. J Cereb Blood Flow Metab. 2021;41(1):105–15. doi: 10.1177/0271678X20902187 31986957 PMC7747167

[93] Xia J, Liu C, Li J, Meng Y, Yang S, Chen H, et al. Decomposing cortical activity through neuronal tracing connectome-eigenmodes in marmosets. Nat Commun. 2024;15(1):2289. doi: 10.1038/s41467-024-46651-8 38480767 PMC10937940

[94] Nakagawa S, Schielzeth H. A general and simple method for obtaining R 2 from generalized linear mixed‐effects models. Methods Ecol Evol. 2012;4(2):133–42. doi: 10.1111/j.2041-210x.2012.00261.x

[95] Brown JA, Lee AJ, Fernhoff K, Pistone T, Pasquini L, Wise AB, et al. Functional network collapse in neurodegenerative disease. Nat Commun. 2025;16(1):10273. doi: 10.1038/s41467-025-65156-6 41271684 PMC12639071

[96] Archer JA, Lee A, Qiu A, Chen S-HA. A comprehensive analysis of connectivity and aging over the adult life span. Brain Connect. 2016;6(2):169–85. doi: 10.1089/brain.2015.0345 26652914

[97] Geerligs L, Renken RJ, Saliasi E, Maurits NM, Lorist MM. A Brain-wide study of age-related changes in functional connectivity. Cereb Cortex. 2015;25(7):1987–99. doi: 10.1093/cercor/bhu012 24532319

[98] He X, Qin W, Liu Y, Zhang X, Duan Y, Song J, et al. Abnormal salience network in normal aging and in amnestic mild cognitive impairment and Alzheimer’s disease. Hum Brain Mapp. 2014;35(7):3446–64. doi: 10.1002/hbm.22414 24222384 PMC6869630

[99] Onoda K, Ishihara M, Yamaguchi S. Decreased functional connectivity by aging is associated with cognitive decline. J Cogn Neurosci. 2012;24(11):2186–98. doi: 10.1162/jocn_a_00269 22784277

[100] Voss MW, Wong CN, Baniqued PL, Burdette JH, Erickson KI, Prakash RS, et al. Aging brain from a network science perspective: something to be positive about?. PLoS One. 2013;8(11):e78345. doi: 10.1371/journal.pone.0078345 24223147 PMC3819386

[101] Abe O, Yamasue H, Aoki S, Suga M, Yamada H, Kasai K, et al. Aging in the CNS: comparison of gray/white matter volume and diffusion tensor data. Neurobiol Aging. 2008;29(1):102–16. doi: 10.1016/j.neurobiolaging.2006.09.003 17023094

[102] Resnick SM, Pham DL, Kraut MA, Zonderman AB, Davatzikos C. Longitudinal magnetic resonance imaging studies of older adults: a shrinking brain. J Neurosci. 2003;23(8):3295–301. doi: 10.1523/JNEUROSCI.23-08-03295.2003 12716936 PMC6742337

[103] Taki Y, Thyreau B, Kinomura S, Sato K, Goto R, Wu K, et al. A longitudinal study of age- and gender-related annual rate of volume changes in regional gray matter in healthy adults. Hum Brain Mapp. 2013;34(9):2292–301. doi: 10.1002/hbm.22067 22438299 PMC6870527

[104] Goulden N, Khusnulina A, Davis NJ, Bracewell RM, Bokde AL, McNulty JP, et al. The salience network is responsible for switching between the default mode network and the central executive network: replication from DCM. Neuroimage. 2014;99:180–90. doi: 10.1016/j.neuroimage.2014.05.052 24862074

[105] Menon V. Large-scale brain networks and psychopathology: a unifying triple network model. Trends Cogn Sci. 2011;15(10):483–506. doi: 10.1016/j.tics.2011.08.003 21908230

[106] Sridharan D, Levitin DJ, Menon V. A critical role for the right fronto-insular cortex in switching between central-executive and default-mode networks. Proc Natl Acad Sci U S A. 2008;105(34):12569–74. doi: 10.1073/pnas.0800005105 18723676 PMC2527952

[107] Dosenbach NUF, Visscher KM, Palmer ED, Miezin FM, Wenger KK, Kang HC, et al. A core system for the implementation of task sets. Neuron. 2006;50(5):799–812. doi: 10.1016/j.neuron.2006.04.031 16731517 PMC3621133

[108] Dajani DR, Uddin LQ. Demystifying cognitive flexibility: Implications for clinical and developmental neuroscience. Trends Neurosci. 2015;38(9):571–8. doi: 10.1016/j.tins.2015.07.003 26343956 PMC5414037

[109] Müller VI, Langner R, Cieslik EC, Rottschy C, Eickhoff SB. Interindividual differences in cognitive flexibility: influence of gray matter volume, functional connectivity and trait impulsivity. Brain Struct Funct. 2015;220(4):2401–14. doi: 10.1007/s00429-014-0797-6 24878823 PMC4981636

[110] Chen T, Cai W, Ryali S, Supekar K, Menon V. Distinct global brain dynamics and spatiotemporal organization of the salience network. PLoS Biol. 2016;14(6):e1002469. doi: 10.1371/journal.pbio.1002469 27270215 PMC4896426

[111] Bonnelle V, Ham TE, Leech R, Kinnunen KM, Mehta MA, Greenwood RJ, et al. Salience network integrity predicts default mode network function after traumatic brain injury. Proc Natl Acad Sci U S A. 2012;109(12):4690–5. doi: 10.1073/pnas.1113455109 22393019 PMC3311356

[112] Jilka SR, Scott G, Ham T, Pickering A, Bonnelle V, Braga RM, et al. Damage to the salience network and interactions with the default mode network. J Neurosci. 2014;34(33):10798–807. doi: 10.1523/JNEUROSCI.0518-14.2014 25122883 PMC4131006

[113] Raichle ME, MacLeod AM, Snyder AZ, Powers WJ, Gusnard DA, Shulman GL. A default mode of brain function. Proc Natl Acad Sci U S A. 2001;98(2):676–82. doi: 10.1073/pnas.98.2.676 11209064 PMC14647

[114] Tomasi D, Volkow ND. Aging and functional brain networks. Mol Psychiatry. 2012;17(5):471, 549–58. doi: 10.1038/mp.2011.81 21727896 PMC3193908

[115] Li X, Salami A, Persson J. Hub architecture of the human structural connectome: Links to aging and processing speed. Neuroimage. 2023;278:120270. doi: 10.1016/j.neuroimage.2023.120270 37423273

[116] Thomas C, Ye FQ, Irfanoglu MO, Modi P, Saleem KS, Leopold DA, et al. Anatomical accuracy of brain connections derived from diffusion MRI tractography is inherently limited. Proc Natl Acad Sci U S A. 2014;111(46):16574–9. doi: 10.1073/pnas.1405672111 25368179 PMC4246325

[117] Hagmann P, Cammoun L, Gigandet X, Meuli R, Honey CJ, Wedeen VJ, et al. Mapping the structural core of human cerebral cortex. PLoS Biol. 2008;6(7):e159. doi: 10.1371/journal.pbio.0060159 18597554 PMC2443193

[118] van den Heuvel MP, Sporns O. Network hubs in the human brain. Trends Cogn Sci. 2013;17(12):683–96. doi: 10.1016/j.tics.2013.09.012 24231140

[119] Grady C. The cognitive neuroscience of ageing. Nat Rev Neurosci. 2012;13(7):491–505. doi: 10.1038/nrn3256 22714020 PMC3800175

[120] Park DC, Reuter-Lorenz P. The adaptive brain: aging and neurocognitive scaffolding. Annu Rev Psychol. 2009;60:173–96. doi: 10.1146/annurev.psych.59.103006.093656 19035823 PMC3359129

[121] Gu S, Pasqualetti F, Cieslak M, Telesford QK, Yu AB, Kahn AE, et al. Controllability of structural brain networks. Nat Commun. 2015;6:8414. doi: 10.1038/ncomms9414 26423222 PMC4600713

[122] Stiernman LJ, Grill F, Hahn A, Rischka L, Lanzenberger R, Panes Lundmark V, et al. Dissociations between glucose metabolism and blood oxygenation in the human default mode network revealed by simultaneous PET-fMRI. Proc Natl Acad Sci U S A. 2021;118(27):e2021913118. doi: 10.1073/pnas.2021913118 34193521 PMC8271663

[123] Gauthier CJ, Madjar C, Desjardins-Crépeau L, Bellec P, Bherer L, Hoge RD. Age dependence of hemodynamic response characteristics in human functional magnetic resonance imaging. Neurobiol Aging. 2013;34(5):1469–85. doi: 10.1016/j.neurobiolaging.2012.11.002 23218565

[124] West KL, Zuppichini MD, Turner MP, Sivakolundu DK, Zhao Y, Abdelkarim D, et al. BOLD hemodynamic response function changes significantly with healthy aging. Neuroimage. 2019;188:198–207. doi: 10.1016/j.neuroimage.2018.12.012 30529628 PMC6450381

