# Supplementary material for: Altered salience network structure–function integration underlies the decline in cognitive flexibility during aging
Source: PLoS Biol. 2026 Apr 13;24(4):e3003738. doi: 10.1371/journal.pbio.3003738 (PMC13089881; doi:10.1371/journal.pbio.3003738)
Supplement: S1 Appendix — Table A. Age and time effects for structure–function coupling and decoupling of all networks of interest in main dataset. Table B. Age and time effects for structure–function coupling and decoupling of all networks of interest in validation dataset. Table C. Association between changes in structure–function coupling/decoupling and cognitive flexibility scores in main and validation datasets. Table D. Sensitivity analysis without global signal regression (GSR): Age and time effects for structure–function coupling and decoupling in main dataset. Table E. Time effects for structure–function coupling and decoupling in main dataset (including sensorimotor networks). Table F. Age and time effects for BOLD activity in main dataset. Fig A. Longitudinal changes in structure–function coupling and decoupling with aging and their associations with cognitive flexibility decline in the main dataset (validation analysis using a 416-ROI parcellation). Fig B. Longitudinal changes in structure–function integration with aging and the associations with cognitive flexibility decline in the main dataset (sensitivity analysis using the L2-norm to quantify component magnitude). Fig C. ROI-level longitudinal changes in structure–function integration in the main dataset (exploratory analysis within the DMN, ECN, and SN). (DOCX) [file pbio.3003738.s001.docx]

**Table A.** Age and time effects for structure-function coupling and decoupling of all networks of interest in main dataset.

| **Effect** | **Signal type** | **Network** | **80% variance** | | | | **90% variance** | | | | **95% variance** | | | |
| --- | --- | --- | --- | --- | --- | --- | --- | --- | --- | --- | --- | --- | --- | --- |
|  |  |  | Estimate | *p* | *FDR-p* | *Bonferroni*  *-p* | Estimate | *p* | *FDR-p* | *Bonferroni-p* | Estimate | *p* | *FDR-p* | *Bonferroni-p* |
| **Time** | Coupled | ECN-A | -0.16 | 0.472 | 0.750 | 1.000 | -0.23 | 0.214 | 0.343 | 1.000 | -0.17 | 0.356 | 0.459 | 1.000 |
|  |  | ECN-B | -0.09 | 0.656 | 0.750 | 1.000 | -0.06 | 0.752 | 0.854 | 1.000 | -0.15 | 0.431 | 0.459 | 1.000 |
|  |  | **ECN-C** | **1.16** | **0.001*** | 0.003* | 0.019* | **0.83** | **0.020*** | 0.081 | 0.325 | **0.82** | **0.018*** | 0.048* | 0.285 |
|  |  | **DMN-A** | **1.46** | **<0.001*** | <0.001* | <0.001* | **1.28** | **<0.001*** | <0.001* | <0.001* | **1.39** | **<0.001*** | <0.001* | <0.001* |
|  |  | DMN-B | 0.01 | 0.960 | 0.960 | 1.000 | -0.21 | 0.298 | 0.373 | 1.000 | -0.17 | 0.428 | 0.459 | 1.000 |
|  |  | DMN-C | 0.16 | 0.656 | 0.750 | 1.000 | 0.06 | 0.854 | 0.854 | 1.000 | 0.09 | 0.806 | 0.806 | 1.000 |
|  |  | **SN-A** | **-0.54** | **<0.001*** | 0.003* | 0.006* | **-0.45** | **0.013*** | 0.069 | 0.207 | **-0.34** | **0.027*** | 0.071 | 0.428 |
|  |  | SN-B | -0.36 | 0.073 | 0.097 | 1.000 | -0.24 | 0.217 | 0.343 | 1.000 | -0.19 | 0.292 | 0.459 | 1.000 |
|  | De-coupled | ECN-A | -0.02 | 0.918 | 0.960 | 1.000 | 0.09 | 0.589 | 0.629 | 1.000 | 0.07 | 0.684 | 0.731 | 1.000 |
|  |  | ECN-B | 0.20 | 0.150 | 0.240 | 1.000 | 0.12 | 0.418 | 0.477 | 1.000 | 0.15 | 0.377 | 0.459 | 1.000 |
|  |  | **ECN-C** | **-1.04** | **0.002*** | 0.003* | 0.026* | **-1.00** | **0.005*** | 0.041* | 0.082 | **-0.93** | **0.009*** | 0.035* | 0.138 |
|  |  | **DMN-A** | **-1.12** | **<0.001*** | <0.001* | <0.001* | **-1.25** | **<0.001*** | <0.001* | <0.001* | **-1.40** | **<0.001*** | <0.001* | <0.001* |
|  |  | DMN-B | 0.27 | 0.192 | 0.256 | 1.000 | 0.25 | 0.254 | 0.373 | 1.000 | 0.35 | 0.091 | 0.145 | 1.000 |
|  |  | DMN-C | 0.38 | 0.109 | 0.217 | 1.000 | 0.38 | 0.132 | 0.263 | 1.000 | 0.46 | 0.086 | 0.145 | 1.000 |
|  |  | **SN-A** | **0.38** | **0.035*** | 0.065 | 0.552 | **0.47** | **0.008*** | 0.067 | 0.134 | **0.43** | **0.008*** | 0.035* | 0.130 |
|  |  | SN-B | 0.18 | 0.182 | 0.256 | 1.000 | 0.19 | 0.147 | 0.263 | 1.000 | 0.22 | 0.111 | 0.148 | 1.000 |
| **Age** | Coupled | **ECN-A** | **-0.32** | **0.010*** | 0.163 | 0.164 | **-0.36** | **0.003*** | 0.055 | 0.055 | **-0.34** | **0.007*** | 0.037* | 0.106 |
|  |  | ECN-B | 0.14 | 0.284 | 0.473 | 1.000 | 0.14 | 0.250 | 0.364 | 1.000 | 0.08 | 0.482 | 0.670 | 1.000 |
|  |  | ECN-C | 0.28 | 0.123 | 0.229 | 1.000 | 0.31 | 0.067 | 0.135 | 1.000 | 0.30 | 0.085 | 0.227 | 1.000 |
|  |  | DMN-A | 0.23 | 0.124 | 0.229 | 1.000 | 0.27 | 0.032* | 0.103 | 0.517 | 0.24 | 0.061 | 0.209 | 0.975 |
|  |  | DMN-B | 0.19 | 0.085 | 0.226 | 1.000 | 0.20 | 0.051 | 0.135 | 0.813 | 0.14 | 0.151 | 0.302 | 1.000 |
|  |  | DMN-C | 0.29 | 0.112 | 0.229 | 1.000 | 0.26 | 0.124 | 0.213 | 1.000 | 0.14 | 0.439 | 0.670 | 1.000 |
|  |  | SN-A | 0.12 | 0.174 | 0.278 | 1.000 | 0.05 | 0.607 | 0.704 | 1.000 | 0.12 | 0.224 | 0.412 | 1.000 |
|  |  | SN-B | 0.04 | 0.615 | 0.789 | 1.000 | 0.10 | 0.224 | 0.344 | 1.000 | 0.11 | 0.121 | 0.302 | 1.000 |
|  | De-coupled | **ECN-A** | **0.23** | **0.030*** | 0.241 | 0.482 | **0.23** | **0.029*** | 0.103 | 0.456 | **0.28** | **0.005*** | 0.037* | 0.085 |
|  |  | ECN-B | -0.01 | 0.932 | 0.932 | 1.000 | -0.04 | 0.719 | 0.863 | 1.000 | -0.01 | 0.913 | 0.913 | 1.000 |
|  |  | ECN-C | -0.20 | 0.227 | 0.453 | 1.000 | -0.22 | 0.187 | 0.324 | 1.000 | -0.23 | 0.176 | 0.321 | 1.000 |
|  |  | DMN-A | -0.23 | 0.054 | 0.217 | 0.866 | -0.27 | 0.025* | 0.103 | 0.405 | -0.32 | 0.009* | 0.037* | 0.147 |
|  |  | DMN-B | -0.13 | 0.164 | 0.278 | 1.000 | -0.13 | 0.162 | 0.323 | 1.000 | -0.07 | 0.473 | 0.670 | 1.000 |
|  |  | DMN-C | -0.01 | 0.896 | 0.932 | 1.000 | -0.02 | 0.863 | 0.863 | 1.000 | -0.09 | 0.428 | 0.670 | 1.000 |
|  |  | SN-A | -0.06 | 0.422 | 0.650 | 1.000 | -0.03 | 0.658 | 0.809 | 1.000 | -0.02 | 0.778 | 0.829 | 1.000 |
|  |  | SN-B | -0.06 | 0.335 | 0.536 | 1.000 | -0.07 | 0.264 | 0.375 | 1.000 | -0.13 | 0.031* | 0.131 | 0.491 |

Estimates (*β*) for effects of age and time on structure-function coupling and decoupling from linear mixed modelling in main dataset, controlled for age, gender and education. Estimates are included for all networks of interest. Asterisks (*) indicate significant estimates with uncorrected *p* < 0.05. Networks in bold show estimates that were found to be significant at all three levels of variances.

**Table B.** Age and time effects for structure-function coupling and decoupling of all networks of interest in validation dataset.

| **Effect** | **Signal type** | **Network** | **80% variance** | | | | **90% variance** | | | | **95% variance** | | | |
| --- | --- | --- | --- | --- | --- | --- | --- | --- | --- | --- | --- | --- | --- | --- |
|  |  |  | Estimate | *p* | *FDR-p* | *Bonferroni-p* | Estimate | *p* | *FDR-p* | *Bonferroni-p* | Estimate | *p* | *FDR-p* | *Bonferroni-p* |
| **Time** | Coupled | ECN-A | -0.04 | 0.817 | 0.897 | 1.000 | -0.12 | 0.481 | 0.771 | 1.000 | -0.20 | 0.293 | 0.887 | 1.000 |
|  |  | ECN-B | -0.03 | 0.812 | 0.897 | 1.000 | -0.19 | 0.201 | 0.765 | 1.000 | -0.06 | 0.695 | 0.887 | 1.000 |
|  |  | ECN-C | -0.11 | 0.752 | 0.897 | 1.000 | -0.20 | 0.578 | 0.771 | 1.000 | -0.18 | 0.615 | 0.887 | 1.000 |
|  |  | DMN-A | 0.05 | 0.781 | 0.897 | 1.000 | -0.02 | 0.939 | 0.939 | 1.000 | -0.10 | 0.600 | 0.887 | 1.000 |
|  |  | **DMN-B** | **0.29** | **0.020*** | 0.160 | 0.320 | 0.21 | 0.107 | 0.251 | 1.000 | **0.29** | **0.026*** | 0.208 | 0.416 |
|  |  | DMN-C | 0.34 | 0.220 | 0.469 | 1.000 | 0.40 | 0.110 | 0.251 | 1.000 | 0.04 | 0.888 | 0.947 | 1.000 |
|  |  | **SN-A** | **-0.41** | **0.010*** | 0.160 | 0.160 | **-0.36** | **0.026*** | 0.208 | 0.416 | **-0.35** | **0.016*** | 0.208 | 0.256 |
|  |  | SN-B | 0.06 | 0.641 | 0.897 | 1.000 | -0.02 | 0.896 | 0.939 | 1.000 | 0.09 | 0.426 | 0.887 | 1.000 |
|  | De-coupled | ECN-A | 0.15 | 0.274 | 0.469 | 1.000 | 0.04 | 0.765 | 0.819 | 1.000 | 0.00 | 0.985 | 0.985 | 1.000 |
|  |  | ECN-B | 0.02 | 0.861 | 0.897 | 1.000 | -0.07 | 0.666 | 0.819 | 1.000 | -0.07 | 0.635 | 0.887 | 1.000 |
|  |  | ECN-C | 0.05 | 0.892 | 0.897 | 1.000 | -0.13 | 0.711 | 0.819 | 1.000 | -0.04 | 0.917 | 0.985 | 1.000 |
|  |  | DMN-A | -0.11 | 0.546 | 0.897 | 1.000 | 0.14 | 0.437 | 0.771 | 1.000 | 0.03 | 0.848 | 0.985 | 1.000 |
|  |  | DMN-B | -0.19 | 0.185 | 0.469 | 1.000 | -0.15 | 0.193 | 0.515 | 1.000 | -0.15 | 0.244 | 0.887 | 1.000 |
|  |  | DMN-C | -0.03 | 0.897 | 0.897 | 1.000 | -0.05 | 0.819 | 0.819 | 1.000 | -0.08 | 0.763 | 0.947 | 1.000 |
|  |  | SN-A | 0.08 | 0.582 | 0.897 | 1.000 | 0.29 | 0.058 | 0.464 | 0.928 | 0.28 | 0.072 | 0.576 | 1.000 |
|  |  | SN-B | 0.13 | 0.294 | 0.469 | 1.000 | 0.09 | 0.524 | 0.771 | 1.000 | 0.07 | 0.633 | 0.887 | 1.000 |
| **Age** | Coupled | ECN-A | -0.17 | 0.317 | 0.856 | 1.000 | -0.22 | 0.190 | 0.490 | 1.000 | -0.19 | 0.247 | 0.657 | 1.000 |
|  |  | ECN-B | 0.11 | 0.344 | 0.856 | 1.000 | 0.13 | 0.238 | 0.490 | 1.000 | 0.15 | 0.185 | 0.657 | 1.000 |
|  |  | ECN-C | -0.35 | 0.115 | 0.856 | 1.000 | -0.19 | 0.406 | 0.541 | 1.000 | -0.08 | 0.751 | 0.910 | 1.000 |
|  |  | DMN-A | 0.16 | 0.332 | 0.856 | 1.000 | 0.20 | 0.209 | 0.490 | 1.000 | 0.18 | 0.263 | 0.657 | 1.000 |
|  |  | DMN-B | 0.09 | 0.387 | 0.856 | 1.000 | 0.08 | 0.490 | 0.541 | 1.000 | -0.01 | 0.940 | 0.985 | 1.000 |
|  |  | DMN-C | -0.25 | 0.199 | 0.856 | 1.000 | -0.14 | 0.421 | 0.541 | 1.000 | -0.17 | 0.330 | 0.704 | 1.000 |
|  |  | SN-A | -0.10 | 0.252 | 0.856 | 1.000 | -0.06 | 0.503 | 0.541 | 1.000 | -0.07 | 0.448 | 0.822 | 1.000 |
|  |  | SN-B | -0.14 | 0.161 | 0.856 | 1.000 | -0.12 | 0.155 | 0.490 | 1.000 | -0.13 | 0.155 | 0.657 | 1.000 |
|  | De-coupled | ECN-A | 0.15 | 0.201 | 0.856 | 1.000 | 0.07 | 0.588 | 0.735 | 1.000 | 0.05 | 0.713 | 0.910 | 1.000 |
|  |  | **ECN-B** | **-0.21** | **0.031*** | 0.496 | 0.496 | **-0.20** | **0.033*** | 0.528 | 0.528 | -0.15 | 0.099 | 0.792 | 1.000 |
|  |  | ECN-C | 0.04 | 0.859 | 0.903 | 1.000 | 0.07 | 0.760 | 0.868 | 1.000 | 0.03 | 0.891 | 0.985 | 1.000 |
|  |  | DMN-A | -0.05 | 0.726 | 0.903 | 1.000 | -0.10 | 0.452 | 0.651 | 1.000 | -0.08 | 0.583 | 0.910 | 1.000 |
|  |  | DMN-B | -0.01 | 0.903 | 0.903 | 1.000 | 0.00 | 0.963 | 0.963 | 1.000 | 0.00 | 0.985 | 0.985 | 1.000 |
|  |  | DMN-C | 0.03 | 0.771 | 0.903 | 1.000 | 0.01 | 0.933 | 0.963 | 1.000 | 0.08 | 0.512 | 0.910 | 1.000 |
|  |  | SN-A | 0.04 | 0.672 | 0.903 | 1.000 | 0.04 | 0.707 | 0.868 | 1.000 | 0.07 | 0.463 | 0.910 | 1.000 |
|  |  | SN-B | 0.01 | 0.856 | 0.903 | 1.000 | 0.04 | 0.641 | 0.855 | 1.000 | 0.04 | 0.641 | 0.910 | 1.000 |

Estimates (*β*) for effects of age and time on structure-function coupling and decoupling from linear mixed modelling in validation dataset, controlled for age, gender and education. Estimates are included for all networks of interest. Asterisks (*) indicate significant estimates with uncorrected *p* < 0.05. Networks in bold show estimates that were found to be significant across at least two levels of variances.

**Table C.** Association between changes in structure-function coupling/decoupling and cognitive flexibility scores in main and validation datasets.

| **Signal type** | **Network** | **Main dataset** | | | | | | **Validation dataset** | | | | | | | |
| --- | --- | --- | --- | --- | --- | --- | --- | --- | --- | --- | --- | --- | --- | --- | --- |
|  |  | **80% var.** | | **90% var.** | | **95% var.** | | **80% var.** | | | **90% var.** | | | **95% var.** | |
|  |  | *b_1_* | *p* | *b_1_* | *p* | *b_1_* | *p* | *b_1_* | *p* | *b_1_* | | *p* | *b_1_* | | *p* |
| Coupled | ECN-C | -0.30 | 0.013 | -0.27 | 0.114 | -0.26 | 0.148 | -2.12 | 0.051 | **2.98** | | **0.010** | **1.34** | | **0.027** |
|  | DMN-A | 0.33 | 0.099 | **0.28** | **0.044** | 0.28 | 0.088 | **-3.89** | **0.009** | 1.13 | | 0.567 | -1.04 | | 0.675 |
|  | DMN-B | **4.23** | **<0.001** | **7.48** | **<0.001** | **-4.29** | **0.012** | **-58.08** | **0.001** | **9.38** | | **0.011** | **-1.97** | | **0.003** |
|  | SN-A | **1.74** | **<0.001** | **0.66** | **0.021** | **1.69** | **<0.001** | 0.93 | 0.119 | **1.62** | | **0.014** | **2.02** | | **0.013** |
| De-coupled | ECN-C | **0.84** | **0.001** | **0.53** | **0.007** | 0.36 | 0.048 | **-2.46** | **0.001** | -1.66 | | 0.059 | **-2.86** | | **0.001** |
|  | DMN-A | -0.54 | 0.011 | **-0.49** | **0.007** | **-0.44** | **0.013** | **2.93** | **0.001** | -8.61 | | 0.118 | 2.12 | | 0.579 |
|  | SN-A | **-0.77** | **0.001** | **-0.63** | **0.005** | **-0.84** | **0.002** | -0.85 | 0.537 | **-2.77** | | **0.002** | **-4.08** | | **0.003** |

Estimates (*b_1_*) for effects of change in structure-function coupling and decoupling on change in TMT (main dataset) or CTT (validation dataset) performance from linear modelling. Estimates are included for all networks with significant time effects from linear mixed modelling analysis. Significant estimates with uncorrected *p* < 0.05 are indicated in bold.

**Table D.** Sensitivity analysis without global signal regression (GSR): Age and time effects for structure-function coupling and decoupling in main dataset.

| **Effect** | **Signal type** | **Network** | **80% variance** | | | **90% variance** | | **95% variance** | | |
| --- | --- | --- | --- | --- | --- | --- | --- | --- | --- | --- |
|  |  |  | Estimate | | *p* | Estimate | *p* | | Estimate | *p* |
| **Time** | Coupled | ECN-A | -0.16 | 0.472 | | -0.23 | 0.214 | | -0.17 | 0.356 |
|  |  | ECN-B | -0.09 | 0.656 | | -0.06 | 0.752 | | -0.15 | 0.431 |
|  |  | **ECN-C** | **1.16** | **0.001*** | | **0.83** | **0.020*** | | **0.82** | **0.018*** |
|  |  | **DMN-A** | **1.46** | **<0.001*** | | **1.28** | **<0.001*** | | **1.39** | **<0.001*** |
|  |  | DMN-B | 0.01 | 0.960 | | -0.21 | 0.298 | | -0.17 | 0.428 |
|  |  | DMN-C | 0.16 | 0.656 | | 0.06 | 0.854 | | 0.09 | 0.806 |
|  |  | **SN-A** | **-0.54** | **<0.001*** | | **-0.45** | **0.013*** | | **-0.34** | **0.027*** |
|  |  | SN-B | -0.36 | 0.073 | | -0.24 | 0.217 | | -0.19 | 0.292 |
|  | Decoupled | ECN-A | -0.02 | 0.918 | | 0.09 | 0.589 | | 0.07 | 0.684 |
|  |  | ECN-B | 0.20 | 0.150 | | 0.12 | 0.418 | | 0.15 | 0.377 |
|  |  | **ECN-C** | **-1.04** | **0.002*** | | **-1.00** | **0.005*** | | **-0.93** | **0.009*** |
|  |  | **DMN-A** | **-1.12** | **<0.001*** | | **-1.25** | **<0.001*** | | **-1.40** | **<0.001*** |
|  |  | DMN-B | 0.27 | 0.192 | | 0.25 | 0.254 | | 0.35 | 0.091 |
|  |  | DMN-C | 0.38 | 0.109 | | 0.38 | 0.132 | | 0.46 | 0.086 |
|  |  | **SN-A** | **0.38** | **0.035*** | | **0.47** | **0.008*** | | **0.43** | **0.008*** |
|  |  | SN-B | 0.18 | 0.182 | | 0.19 | 0.147 | | 0.22 | 0.111 |
| **Age** | Coupled | **ECN-A** | **-0.32** | **0.010*** | | **-0.36** | **0.003*** | | **-0.34** | **0.007*** |
|  |  | ECN-B | 0.14 | 0.284 | | 0.14 | 0.250 | | 0.08 | 0.482 |
|  |  | ECN-C | 0.28 | 0.123 | | 0.31 | 0.067 | | 0.30 | 0.085 |
|  |  | DMN-A | 0.23 | 0.124 | | 0.27 | 0.032* | | 0.24 | 0.061 |
|  |  | DMN-B | 0.19 | 0.085 | | 0.20 | 0.051 | | 0.14 | 0.151 |
|  |  | DMN-C | 0.29 | 0.112 | | 0.26 | 0.124 | | 0.14 | 0.439 |
|  |  | SN-A | 0.12 | 0.174 | | 0.05 | 0.607 | | 0.12 | 0.224 |
|  |  | SN-B | 0.04 | 0.615 | | 0.10 | 0.224 | | 0.11 | 0.121 |
|  | Decoupled | **ECN-A** | **0.23** | **0.030*** | | **0.23** | **0.029*** | | **0.28** | **0.005*** |
|  |  | ECN-B | -0.01 | 0.932 | | -0.04 | 0.719 | | -0.01 | 0.913 |
|  |  | ECN-C | -0.20 | 0.227 | | -0.22 | 0.187 | | -0.23 | 0.176 |
|  |  | DMN-A | -0.23 | 0.054 | | -0.27 | 0.025* | | -0.32 | 0.009* |
|  |  | DMN-B | -0.13 | 0.164 | | -0.13 | 0.162 | | -0.07 | 0.473 |
|  |  | DMN-C | -0.01 | 0.896 | | -0.02 | 0.863 | | -0.09 | 0.428 |
|  |  | SN-A | -0.06 | 0.422 | | -0.03 | 0.658 | | -0.02 | 0.778 |
|  |  | SN-B | -0.06 | 0.335 | | -0.07 | 0.264 | | -0.13 | 0.031* |

Estimates (*β*) for effects of age and time on structure-function coupling and decoupling from linear mixed modelling in main dataset, controlled for age, gender and education. Estimates are included for all networks of interest. Asterisks (*) indicate significant estimates with uncorrected *p* < 0.05. Networks in bold show estimates that were found to be significant at all three levels of variances.

**Table E.** Time effects for structure-function coupling and decoupling in main dataset (including sensorimotor networks).

| **Effect** | **Signal type** | **Network** | **Estimate** | ***Raw p*** | ***FDR-p*** | ***Bonferroni-p*** |
| --- | --- | --- | --- | --- | --- | --- |
| **Time** | Coupled | ECN-A | -0.17 | 0.356 | 0.512 | 1.000 |
|  |  | ECN-B | -0.15 | 0.431 | 0.512 | 1.000 |
|  |  | ECN-C | 0.82 | 0.018* | 0.056 | 0.339 |
|  |  | **DMN-A** | **1.39** | **<0.001*** | **<0.001*** | **<0.001*** |
|  |  | DMN-B | -0.17 | 0.428 | 0.512 | 1.000 |
|  |  | DMN-C | 0.09 | 0.806 | 0.806 | 1.000 |
|  |  | SN-A | -0.34 | 0.027* | 0.073 | 0.509 |
|  |  | SN-B | -0.19 | 0.292 | 0.463 | 1.000 |
|  |  | SomMot | 0.45 | 0.039***** | 0.093 | 0.741 |
|  |  | Visual | 0.07 | 0.617 | 0.690 | 1.000 |
|  | Decoupled | ECN-A | 0.07 | 0.684 | 0.722 | 1.000 |
|  |  | ECN-B | 0.15 | 0.377 | 0.512 | 1.000 |
|  |  | **ECN-C** | **-0.93** | **0.009*** | **0.041*** | 0.164 |
|  |  | **DMN-A** | **-1.40** | **<0.001*** | **<0.001*** | **<0.001*** |
|  |  | DMN-B | 0.35 | 0.091 | 0.172 | 1.000 |
|  |  | DMN-C | 0.46 | 0.086 | 0.172 | 1.000 |
|  |  | **SN-A** | **0.43** | **0.008*** | **0.041*** | 0.155 |
|  |  | SN-B | 0.22 | 0.111 | 0.192 | 1.000 |
|  |  | SomMot | -0.43 | 0.016***** | 0.056 | 0.300 |
|  |  | Visual | -0.33 | 0.356 | 0.512 | 1.000 |

Estimates (*β*) for effects of time on structure-function coupling and decoupling from linear mixed modelling in main dataset, controlled for age, gender and education. Estimates are included for all networks of interest. Asterisks (*) indicate significant estimates with uncorrected *p* < 0.05. Networks in bold show estimates that were found to be significant after FDR or Bonferroni correction.

**Table F.** Age and time effects for BOLD activity in main dataset.

| **Effect** | **Network** | **Estimate** | ***p*** | **Effect** | **Network** | **Estimate** | ***p*** |
| --- | --- | --- | --- | --- | --- | --- | --- |
| **Time** | ECN-A | 0.0033 | 0.197 | **Age** | ECN-A | 0.0019 | 0.207 |
|  | ECN-B | 0.0034 | 0.339 |  | ECN-B | 0.0004 | 0.826 |
|  | ECN-C | 0.0000 | 0.225 |  | ECN-C | 0.0000 | 0.925 |
|  | DMN-A | 0.0000 | 0.703 |  | DMN-A | 0.0000 | 0.362 |
|  | DMN-B | 0.2446 | 0.011* |  | DMN-B | 0.0957 | 0.109 |
|  | DMN-C | -0.0196 | 0.313 |  | DMN-C | -0.0001 | 0.809 |
|  | SN-A | -0.0570 | 0.025* |  | SN-A | 0.0028 | 0.166 |
|  | SN-B | -0.0075 | 0.172 |  | SN-B | -0.0003 | 0.718 |

Estimates (*β*) for effects of age and time on BOLD activity from linear mixed modelling in main dataset, controlled for age, gender and education. Estimates are included for all networks of interest. Asterisks (*) indicate significant estimates with uncorrected *p* < 0.05.

**

**

**Fig A**. **Longitudinal changes in structure–function coupling and decoupling with aging and their associations with cognitive flexibility decline in the main dataset (validation analysis using a 416-ROI parcellation).** **A & B.** Longitudinal effects were estimated using linear mixed-effects models, with fixed coefficients (β) representing the effect of time interval from the first scan. Spaghetti plots display individual trajectories of structure–function coupling and decoupling over time, adjusted for baseline age, gender, and education. Coupling and decoupling measures were computed using *K* values explaining 95% of variance, shown here for illustration. Brain network visualizations were created using BrainNet Viewer (<http://www.nitrc.org/projects/bnv>). **C.** Associations between longitudinal change rates in cognitive flexibility, measured by the Trail Making Test (TMT) and change rates in structure–function integration are shown. Scatter plots illustrate the relationship between re-estimated individual slopes (*β*_1j_) in TMT (or CTT) performance and coupling/decoupling metrics, adjusted for baseline age, gender and education.





**Fig B**. **Longitudinal changes in structure–function integration with aging and the associations with cognitive flexibility decline in the main dataset (sensitivity analysis using the L2-norm to quantify component magnitude).** **A & B.** Longitudinal effects were estimated using linear mixed-effects models, with fixed coefficients (β) representing the effect of time interval from the first scan. Spaghetti plots display individual trajectories of structure–function coupling and decoupling over time, adjusted for baseline age, gender, and education. Coupling and decoupling metrics were computed using the same variance-based component selection criterion (K values explaining 95% of the signal variance), and were summarized using the L2-norm to quantify component magnitude. **C.** Associations between longitudinal change rates in cognitive flexibility, measured by the Trail Making Test (TMT) and change rates in structure–function integration are shown. Scatter plots illustrate the relationship between re-estimated individual slopes (*β*_1j_) in TMT (or CTT) performance and coupling/decoupling metrics, adjusted for baseline age, gender and education.





**Fig C**. **ROI-level longitudinal changes in structure–function integration in the main dataset (exploratory analysis within the DMN, ECN, and SN).** Longitudinal effects were estimated using linear mixed-effects models, with fixed coefficients (β) representing the effect of time interval from the first scan. Spaghetti plots display individual trajectories of ROI-level structure–function coupling over time, adjusted for baseline age, gender, and education. Coupling and decoupling metrics were computed using the same variance-based component selection criterion (K values explaining 95% of the signal variance). ROI-level p-values were corrected for multiple comparisons using Bonferroni correction across ROIs within these networks. **Abbreviations:** LH = left hemisphere; RH = right hemisphere; SN = salience network; DMN = default mode network; SalVentAttn = salience/ventral attention network; A/B = subnetwork A/B; DefaultA = default mode subnetwork A; FrMed = frontal medial cortex; PFCm = medial prefrontal cortex; PFCd = dorsolateral prefrontal cortex.
