# Supplementary material for: Altered salience network structure–function integration underlies the decline in cognitive flexibility during aging
Source: PLoS Biol. 2026 Apr 13;24(4):e3003738. doi: 10.1371/journal.pbio.3003738 (PMC13089881; doi:10.1371/journal.pbio.3003738)
Supplement: Attachment — Submitted filename: Response_to_reviewer_v2.docx [file pbio.3003738.s007.docx]

**Responses to the Reviewers**

**Reviewer #1:** Cognitive flexibility is a fundamental component of executive function. Cognitive flexibility relies on the integration between brain functional dynamics and structural architecture. This study integrated multimodal MRI data with advanced graph signal processing to address a key question about how the structural architecture constrains functional dynamics in aging. Overall, this work is interesting, but several major and minor concerns remain.

1. The Abstract and Introduction are somewhat redundant and could be more concise. Currently, both sections contain overlapping background information and methodological descriptions, which obscure the study's key message.

**Response:** We thank the reviewer for this helpful comment. In response, we have revised both the Abstract and Introduction to reduce redundancy and improve conciseness.

Specifically, we streamlined the Abstract by shortening overlapping background statements and methodological descriptions, and by focusing more on the key longitudinal findings and their implications. We revised the Introduction by condensing the general background on cognitive flexibility and age-related structural and functional brain changes, while sharpening the rationale for examining structure–function integration.

These revisions can be found in the **Abstract** and the revised **first four paragraphs of the Introduction** (highlighted in yellow).

2. The authors should clarify the conceptual and methodological distinction between the present work and the previous study (https://www.nature.com/articles/s41562-017-0260-9). Specifically, the manuscript should more clearly articulate how the current longitudinal investigation in aging extends or differs from that prior work in terms of experimental design, analytical framework, and research focus.

**Response:** We thank the reviewer for this important comment. We have now clarified the conceptual and methodological distinctions between the present study and the prior work as follows.

First, the research focus and population differ: the prior study examined structure–function alignment/liberality in young adults during a cognitive switching task. In contrast, the present work studied **cognitively normal older adults** aiming to characterize aging-related changes in intrinsic structure–function alignment/liberality.

Second, the experimental design differs. The previous study was cross-sectional and task-based, focusing on task-evoked dynamics during in-scanner cognitive switching. In contrast, our study uses a **longitudinal** design and quantifies **intrinsic** structure–function integration from resting-state fMRI, allowing us to assess intra-individual changes over time and their relationships with cognitive decline over time.

Third, the behavioral outcomes also differ. The prior work related structure–function integration to in-scanner task performance. Here, we extended it further to link longitudinal changes in **intrinsic** structure–function integration to **out-of-scanner** neuropsychological measures of cognitive flexibility (Trail Making Test and Color Trails Test), which enhances ecological validity and generalizability.

Finally, the present study examines structure–function alignment/liberality at the **network/subnetwork level** (within ECN, DMN, and SN), whereas the previous study reported only whole-brain averaged estimates.

These distinctions have been explicitly stated in the **Introduction**:

- **Introduction (Paragraph 5):** “the present study examined longitudinal changes in intrinsic structure–function integration (coupling and decoupling) at the network level in normal aging …”; “Since most prior structure–function integration studies in aging are cross-sectional, making it difficult to distinguish true within-person change from cohort effects, our longitudinal approach allows us to quantify within-individual trajectories of structure–function coupling/decoupling.”; “Particularly, we assessed intrinsic structure–function integration from resting-state fMRI, providing a task-independent characterization of aging-related changes that complements prior task-based work.”; and “We focused on the subnetworks of ECN, DMN, and SN because they form core large-scale systems …”

3. By applying a graph Fourier transform to decompose resting-state fMRI signals according to the underlying structural connectivity, the authors quantify two complementary indices—alignment and liberality. I would suggest reconsidering the use of the terms "alignment" and "liberality", as they may be somewhat misleading to readers who are not familiar with this specific framework. Using more conventional terminology, such as "coupling" and "decoupling," would improve conceptual clarity and make the findings easier to interpret within the broader context of structure-function integration studies (e.g., https://doi.org/10.1038/s41467-024-46651-8IF: 15.7 Q1 and <https://www.nature.com/articles/s41467-019-12765-7).>

**Response:** We thank the reviewer for this suggestion. We agree that the terms “alignment” and “liberality,” while adopted from the original graph signal processing framework, may be unfamiliar to broader readers. To improve conceptual clarity, as recommended, we have revised the manuscript to explicitly link these measures to more conventional terminology "coupling" and "decoupling".

Specifically, we now refer to alignment as structure–function coupling and liberality as decoupling (and similarly “aligned” and “liberal” as “coupled” and “decoupled”), while retaining the original terms in parentheses at first mention.

In the **Abstract**, we added “(i.e., coupling)” and “(i.e., decoupling)” when the terms first appear, and we use coupling/decoupling terminology throughout the remainder of the text for readability. Importantly, we also added clarifying statements in the **Introduction**, **Methods** and **Discussion** to emphasize that, although conceptually related to coupling/decoupling, these indices are derived from graph Fourier/spectral decomposition of regional BOLD signals with respect to the structural connectome, and thus are distinct from conventional SC–FC correlation-based coupling measures. Finally, we updated **Results, all figures** and **figure legends** accordingly by replacing “alignment” and “liberality” with “coupling” and “decoupling.”

Specifically, we revised/added the following sentences in the main text:

- **Introduction (Paragraph 4):** “Specifically, alignment reflects stronger structural constraints on intrinsic functional dynamics (i.e., higher coupling), whereas liberality reflects greater deviations from structural architecture (i.e., higher decoupling). Importantly, these indices differ from conventional SC–FC coupling measures because they are derived from graph spectral decomposition of BOLD signals with respect to the structural connectome, rather than from correlations between SC and FC. To improve accessibility, we refer to alignment and liberality as coupling and decoupling, respectively, while noting that these terms are defined within the present graph-spectral framework and are distinct from correlation-based coupling/decoupling metrics.”
- **Methods - *Derivation of structure-function coupling and decoupling* (Paragraph 1):** “For clarity and accessibility, we use the terms coupling and decoupling to refer to the alignment and liberality indices in the original study. Importantly, these measures should not be conflated with conventional structure–function coupling metrics based on SC–FC correlations, as they are computed via graph spectral decomposition of BOLD signals with respect to the structural connectome.”
- **Discussion** (**Paragraph 1**): “Our graph-spectral coupling/decoupling metrics are not intended to replicate these established findings; rather, they provide complementary information by quantifying how the structural connectome constrains intrinsic functional dynamics.”

4. The authors employed a network parcellation of 126 regions of interest (ROIs), including 114 cortical regions from Yeo et al. and 12 subcortical regions. Although this atlas is well-established, it would be interesting and valuable to assess the robustness of the reported findings using higher-resolution parcellations. For instance, repeating the key analyses with the HCP-MMP1.0+Tian Subcortex atlas or the Schaefer2018_400Parcels_7Networks_order_Tian_Subcortex_S1 parcellation could provide necessary confirmation of the reproducibility of the observed structure-function relationships.

**Response:** We thank the reviewer for this helpful suggestion. As recommended, we assessed the robustness of our findings by repeating the key analyses in the main dataset using another higher-resolution brain parcellation.

Specifically, we applied an alternative parcellation comprising **416 ROIs**, constructed by combining the Schaefer 400-parcel (17-network) cortical parcellation with the Tian 16-parcel subcortical atlas. Overall, the findings remained largely consistent with the primary results.

Specifically, the SN-A subnetwork exhibited decreased structure–function coupling and increased decoupling over time, and these longitudinal changes were significantly associated with cognitive flexibility decline as measured by the Trail Making Test (TMT). In contrast, the DMN-A subnetwork showed the opposite pattern, with increased coupling and decreased decoupling over time. The corresponding figures are provided in **Supplementary Figure 1**. However, ECN-C did not show significant longitudinal effects under the alternative parcellation (β≈0, p>0.80). This discrepancy may reflect reduced robustness of the ECN-C effect across parcellations, potentially due to differences in how this relatively small subnetwork is represented (e.g., altered spatial boundaries), which may increase measurement noise and reduce sensitivity to detect subtle longitudinal changes. In fact, ECN-C is right next to the DMN subnetwork.

In light of this replication analysis, the validation dataset results (Figure 3), and the ROI-level analysis (Response 7 to Reviewer #1), we have now revised the manuscript to report the most robust findings—those involving SN and DMN—in the main Figure 2. The ECN beta and p values remain available in Supplementary Table 1. Accordingly, we updated the Abstact, Results and Discussion sections to reflect a more cautious conclusion based on the robust SN and DMN effects.

We have revised the Methods, Results and Discussion accordingly, as detailed below:

- **Methods - *Structural network construction and BOLD time series extraction*** **(Paragraph 1):** “To assess the robustness of our findings, we repeated the analyses in the main dataset using an alternative brain parcellation with 416 ROIs, constructed by combining the Schaefer’s 400-parcel (17-network) cortical parcellation with the Tian’s 16-parcel subcortical atlas.”
- **Results - *Longitudinal changes in cognitive flexibility were associated with structure-function coupling and decoupling changes in the salience network*** **(the last paragraph):** “When replicating the analyses in the main dataset using an alternative parcellation comprising 416 ROIs, the overall findings remained largely consistent with the primary results (Supplementary Figure 1). Specifically, the SN-A subnetwork exhibited decreased structure–function coupling and increased decoupling over time, and these longitudinal changes were significantly associated with cognitive flexibility decline as measured by the Trail Making Test (TMT). In contrast, the DMN-A subnetwork showed the opposite pattern, with increased coupling and decreased decoupling over time. However, ECN-C did not show significant longitudinal effects under the alternative parcellation (β≈0, p>0.80). This discrepancy may reflect reduced robustness of the ECN-C effect across parcellations, potentially due to differences in how this relatively small subnetwork is represented (e.g., altered spatial boundaries), which may increase measurement noise and reduce sensitivity to detect subtle longitudinal changes. ”
- **Discussion - *Biological interpretation of structure-function coupling and decoupling in aging* (Paragraph 2)**: “the aging-related increase in coupling and decrease in decoupling observed in DMN-A—robust across the main analysis, sensitivity analyses using a finer parcellation resolution, and ROI-level modeling—may reflect”
- **Discussion - *Differential structure-function integration changes in task-positive and task-negative networks in aging*** **(Paragraph 1)**: “it is plausible that ECN-C shows convergence toward DMN-like organization. Notably, however, this ECN-C effect was smaller in magnitude and was attenuated in sensitivity analyses using a finer parcellation resolution and in ROI-level modeling, suggesting it may be less robust than the DMN-A and SN-A findings”
- **Supplementary Figures**

**Supplementary Figure 1. Longitudinal changes in structure–function coupling and decoupling with aging and their associations with cognitive flexibility decline in the main dataset (validation analysis using a 416-ROI parcellation). A & B.** Longitudinal effects were estimated using linear mixed-effects models, with fixed coefficients (β) representing the effect of time interval from the first scan. Spaghetti plots display individual trajectories of structure–function coupling and decoupling over time, adjusted for baseline age, gender, and education. Coupling and decoupling measures were computed using K values explaining 95% of variance, shown here for illustration. Brain network visualizations were created using BrainNet Viewer (<http://www.nitrc.org/projects/bnv>). **C.** Associations between longitudinal change rates in cognitive flexibility, measured by the Trail Making Test (TMT) and change rates in structure–function integration are shown. Scatter plots illustrate the relationship between re-estimated individual slopes (β1j) in TMT (or CTT) performance and coupling/decoupling metrics, adjusted for baseline age, gender and education.

5. The authors derived the alignment and liberality using a variance-based criterion to determine the cut-off (K_A and K_L) in the structural eigenvalue spectrum. This approach differs from prior studies, which typically employed a spectrum dichotomization method (https://www.nature.com/articles/s41467-019-12765-7) or selected only the highest and lowest eigenmodes (https://www.nature.com/articles/s41562-017-0260-9) to represent smooth and rough components. The authors should clarify the conceptual and methodological advantages of the variance-based criterion over these conventional approaches.

**Response:** We thank the reviewer for this important comment. We have now clarified the rationale and advantages of the variance-based criterion for determining the cut-offs for coupling (alignment) and decoupling (liberality), summarized as follows.

First, the variance-based criterion allows the cut-offs ($K_{A}$ and $K_{L}$) to be data-adaptive rather than imposed a priori. Unlike selecting a fixed number of eigenmodes (e.g., the first/last *N* components), which depends on the ROI count of chosen parcellation, our approach determines $K_{A}$ and $K_{L}$ based on the number of modes required to explain a pre-specified proportion of variance. This yields coupling/decoupling indices that are comparable across parcellations and avoids arbitrary choices of *N*. For example, using the same 90% variance threshold, the 126-ROI parcellation in the main dataset yielded $K_{A}$ = 12 and $K_{L}$ = 66, whereas the 416-ROI parcellation in the validation analysis yielded $K_{A}$ = 52 and $K_{L}$ = 199.

Second, compared with spectrum dichotomization methods that impose a single cut-off frequency (e.g., splitting the spectrum into two halves or using a fixed threshold), the variance-based criterion provides a more consistent definition of low- and high-frequency subspaces across analysis settings. A fixed cut-off may not guarantee that the resulting “low-frequency” and “high-frequency” components capture comparable proportions of spectral energy, whereas the variance-based approach ensures that coupling/decoupling components represent a consistent proportion of signal content.

Accordingly, we added a dedicated clarification in the Methods section to address this point:

- **Methods** ***- Derivation of structure-function coupling and decoupling*** **(Paragraph 4):** “Rather than selecting a fixed number of graph spectral components or applying a spectrum dichotomization threshold as in prior studies [62, 63], we determined the coupling and decoupling components using a variance-based criterion derived from the structural eigenvalue spectrum, which provides a principled way to define low- and high-frequency subspaces and is robust across parcellations with different ROI counts.”

6. The authors averaged the aligned and liberal signal components across time points and ROIs to obtain static, network-level measures of structure-function alignment and liberality. In contrast, previous studies have typically used the L2-norm of the component vectors to summarize their magnitude. The authors should clarify the rationale for choosing a simple averaging approach over the L2-norm, and discuss whether this choice could affect the interpretation of alignment and liberality measures (such as <https://doi.org/10.1038/s41467-024-46651-8> IF: 15.7 Q1 and <https://www.nature.com/articles/s41467-019-12765-7).>

**Response:** We thank the reviewer for this comment. We agree that averaging and the L2-norm summarize different aspects of the aligned (coupled) and liberal (decoupled) component time series: the L2-norm captures overall magnitude/energy, whereas averaging provides an estimate of the mean coupled/decoupled signal contribution while preserving sign**,** and is less influenced by isolated extreme values.

To evaluate whether our findings depend on the choice of summary metric, we now newly conducted a sensitivity analysis using the L2-norm approach. Specifically, we re-computed network-level coupling and decoupling in the main dataset (126-ROI parcellation) using the L2-norm of the coupled/decoupled component vectors. Because the L2-norm is sign-invariant, we applied a constant shift to the component time series to ensure non-negativity and facilitate comparability with the averaging-based measures. Importantly, the longitudinal effects and brain–behavior associations were largely consistent with the main results (Supplementary Figure 2), supporting the robustness of our conclusions.

We have added the following descriptions to the manuscript:

- **Methods** ***- Derivation of structure-function coupling and decoupling*** **(Paragraph 6):** “In addition to averaging the coupled and decoupled signal components across time points to obtain static, network-level measures, we conducted a sensitivity analysis using an alternative summary metric commonly adopted in prior studies [63, 91], namely the L2-norm of the component vectors. Network-level coupling and decoupling were re-computed in the main dataset (126-ROI parcellation) using the L2-norm to quantify component magnitude. Because the L2-norm is sign-invariant, we applied a constant shift to the coupled/decoupled component time series to ensure non-negativity prior to computing the norm.”
- **Results - *Longitudinal changes in cognitive flexibility were associated with structure-function coupling and decoupling changes in the salience network* (Paragraph 3):** “Sensitivity analyses using the L2-norm summarization approach yielded longitudinal effects and brain–behavior associations for structure–function coupling/decoupling that were largely consistent with the main results (Supplementary Figure 2).”
- **Supplementary Figures**

**Supplementary Figure 2. Longitudinal changes in structure–function integration with aging and the associations with cognitive flexibility decline in the main dataset (sensitivity analysis using the L2-norm to quantify component magnitude). A & B.** Longitudinal effects were estimated using linear mixed-effects models, with fixed coefficients (β) representing the effect of time interval from the first scan. Spaghetti plots display individual trajectories of structure–function coupling over time, adjusted for baseline age, gender, and education. Coupling and decoupling metrics were computed using the same variance-based component selection criterion (K values explaining 95% of the signal variance) and were summarized using the L2-norm to quantify component magnitude. **C.** Associations between longitudinal change rates in cognitive flexibility, measured by the Trail Making Test (TMT) and change rates in structure–function integration are shown. Scatter plots illustrate the relationship between re-estimated individual slopes (β1j) in TMT (or CTT) performance and coupling/decoupling metrics, adjusted for baseline age, gender and education.

7. The authors focused on the executive control network (ECN), default mode network (DMN), and salience network (SN) as primary targets for their analyses. However, the rationale for selecting these three networks over others is not fully articulated. Additionally, exploring age-related structure-function integration at the regional level and linking these regional measures to cognitive performance could provide more detailed insights.

**Response:** We thank the reviewer for this helpful comment. We have now expanded the rationale for focusing on the ECN, DMN, and SN. These three networks have been consistently implicated in cognitive flexibility and executive control, show robust age-related alterations in functional segregation and structural connectivity, and are considered core systems supporting dynamic switching between internally and externally oriented cognition. In particular, the SN plays a central role in detecting salient information and coordinating interactions between the ECN and DMN, a mechanism closely linked to cognitive flexibility.

We revised the Introduction accordingly:

- **Introduction** (the last paragraph): “We focused on subnetworks within the ECN, DMN, and SN because these networks form core large-scale systems supporting higher-order cognition, including cognitive flexibility and executive control. They show robust age-related alterations in functional segregation and structural connectivity, and the SN in particular coordinates dynamic switching between the ECN and DMN.”

We thank the reviewer for the suggestions on exploring age-related structure-function integration at the regional level. We agree that ROI-level analyses may provide additional insights by offering finer-grained localization. To address this, we conducted an exploratory ROI-level analysis of structure–function coupling/decoupling within these three networks.

The results are presented in Supplementary Figure 3. Importantly, the ROI-level patterns were broadly consistent with the network-level findings, with the strongest aging-related coupling/decoupling changes observed in SN-A and DMN-A regions (e.g., Salience/Ventral Attention Network A – Frontal Medial region [SalVentAttnA_FrMed], Default Mode Network A – medial prefrontal cortex [DefaultA_PFCm]) after Bonferroni correction, further supporting the robustness of our conclusions. We have added a brief description of this exploratory analysis in the Methods, Results and Discussion sections as follows:

- **Methods -** ***Estimation of aging-related changes in structure-function integration in older adults*** (Paragraph 3): “To provide additional insights, we also conducted an exploratory ROI-level analysis of structure–function coupling/decoupling within the ECN, DMN, and SN subnetworks. For each ROI, longitudinal effects were tested using linear mixed-effects models consistent with the main analyses, and ROI-level p-values were corrected for multiple comparisons using Bonferroni correction across ROIs within these networks.”
- **Results** ***- Network-level structure-function integration showed differential longitudinal changes in older adults*** (Paragraph 6): “Exploratory ROI-level analyses in main dataset revealed patterns broadly consistent with the network-level findings, with the strongest aging-related coupling/decoupling changes observed in ROIs within the SN-A and DMN-A subnetworks (e.g., SalVentAttnA_FrMed and DefaultA_PFCm; Supplementary Figure 3).”
- **Discussion** - ***Maintenance of normative salience network structure-function integration in aging supports cognitive flexibility*** (Paragraph 2): “While ROI-level findings provide finer-grained localization, we interpret them as exploratory given the multiple-comparison burden and potential parcel-level noise; nevertheless, their consistency with network-level results supports the robustness of our main conclusions.”
- **Supplementary Figures**

**Supplementary Figure 3. ROI-level longitudinal changes in structure–function integration in the main dataset (exploratory analysis within the DMN, ECN, and SN).** Longitudinal effects were estimated using linear mixed-effects models, with fixed coefficients (β) representing the effect of time interval from the first scan. Spaghetti plots display individual trajectories of ROI-level structure–function coupling over time, adjusted for baseline age, gender, and education. Coupling and decoupling metrics were computed using the same variance-based component selection criterion (K values explaining 95% of the signal variance). ROI-level p-values were corrected for multiple comparisons using Bonferroni correction across ROIs within these networks. Abbreviations: LH = left hemisphere; RH = right hemisphere; SN = salience network; DMN = default mode network; SalVentAttn = salience/ventral attention network; A/B = subnetwork A/B; DefaultA = default mode subnetwork A; FrMed = frontal medial cortex; PFCm = medial prefrontal cortex; PFCd = dorsolateral prefrontal cortex.

8. The physical meaning of age coefficients (beta) of age-related changes is difficult to comprehend. To provide insight into the overall magnitude and direction of regional age effects, many previous studies have represented the age effects (R-squared values) as the age-related changes during development (see, for example, https://www.nature.com/articles/s41586-022-04554-y and <https://www.nature.com/articles/s41593-025-01907-4>).

**Response:** We thank the reviewer for this comment. In our longitudinal linear mixed-effects models, the primary predictor of interest was time since baseline (visit interval), while chronological age at baseline was included as a covariate. Thus, the reported β coefficients reflect the estimated rate of longitudinal change per unit time, and their sign indicates the direction of change.

As suggested by the reviewer, to provide a more intuitive summary of the magnitude of longitudinal effects, we additionally quantified the variance explained by the time-related fixed effects using a marginal R² framework for mixed-effects models (Nakagawa, 2013). Specifically, we fit a reduced model by removing the time and baseline age × time fixed-effect terms from Equation (5), while keeping the same random-effects structure. We then computed the incremental explained variance as ΔR²m = R²m(full) − R²m(reduced), which represents the additional variance explained by the time-related fixed effects. In parallel, we performed likelihood ratio tests comparing the full and reduced models; a significant model comparison (p < 0.05) indicates that the time-related fixed effects explain a significant amount of variance. We have added these effect-size summaries and model comparison results to the revised manuscript as follows.

- **Methods** - ***Estimation of aging-related changes in structure-function integration in older adults*** (Paragraph 2): “To provide an intuitive summary of the magnitude of longitudinal effects, we additionally quantified the variance explained by the time-related fixed effects using a marginal R² (R²m) framework for mixed-effects models [91]. Specifically, we fit a reduced model by removing the time and baseline age × time fixed-effect terms from Equation (5), while keeping the same random-effects structure. We then computed the incremental explained variance as ΔR²m = R²m(full) − R²m(reduced), which represents the additional variance explained by the time-related fixed effects. In parallel, we performed likelihood ratio tests comparing the full and reduced models. To facilitate interpretation, ΔR²m values were used as effect-size summaries of time-related longitudinal change and were reported alongside β coefficients.”
- **Results** ***- Network-level structure-function integration showed differential longitudinal changes in older adults*** (Paragraph 4): “For structure–function coupling, the additional variance explained by the time-related fixed effects (ΔR²m) was 0.025 for SN-A, 0.127 for DMN-A, and 0.032 for ECN-C. Likelihood ratio tests comparing the full and reduced models yielded p-values of 0.025, < 0.001, and 0.061, respectively. For structure–function decoupling, the corresponding ΔR²m values were 0.052 for SN-A, 0.163 for DMN-A, and 0.049 for ECN-C, with model comparison p-values of 0.022, < 0.001, and 0.033, respectively.”

**Reviewer #2:** This longitudinal study examines brain structure-function integration in aging, focusing on the executive control, default, and salience networks. The authors report differences in alignment between structural and functional network properties of these networks with age and associated them with declines in cognitive flexibility over time. This is an interesting and timely study. Specific comments are below.

1. In the abstract, the term "liberality" is used before it is defined. Overall, it is not clear why the term "liberality" is introduced at all. This is a confusing term, as it is not typically used in describing functional brain networks. The manuscript and findings would be easier to follow if the authors simply used the term "alignment" (e.g. greater or lesser alignment between functional and structural networks). If increased alignment means decreased liberality, then there is no need for two terms. If they are really measuring different things, this should be more clearly spelled out.

**Response:** We thank the reviewer for this comment. We agree that the terms “alignment” and “liberality,” although adopted from the original graph signal processing framework, may be unfamiliar to broader readers. As described in our **Response 3 to Reviewer #1**, we have revised the manuscript to explicitly link these measures to more conventional terminology, referring to alignment as structure–function coupling and liberality as decoupling.

To clarify for the reviewer’s concern regarding why two terms are needed, in this graph-spectral framework, the fMRI signal is decomposed into components that are aligned with the structural connectome (low-frequency/smooth modes) versus components that deviate liberally from the structural architecture (high-frequency/rough modes). Accordingly, we quantify two complementary indices: alignment (coupling), reflecting the degree to which intrinsic functional dynamics are constrained by white matter structure, and liberality (decoupling), reflecting the extent to which functional dynamics deviate from structural constraints. Importantly, these indices are not simply redundant opposites or perfect inverses of each other, because they summarize energy/variance contributions from two distinct spectral subspaces; thus, both can vary independently across networks and age.

To address the reviewer’s suggestion and to enhance accessibility, we made the following revisions:

- We now refer to alignment as structure–function coupling and liberality as structure–function decoupling, while retaining the original terms in parentheses at first mention (e.g., “coupling (alignment)” and “decoupling (liberality)”; see details in **Response 3 to Reviewer #1**).
- We added a brief clarification in the **Abstract/Introduction/Methods** explaining that these measures represent distinct spectral components (aligned/coupled vs liberal/decoupled) and should not be interpreted as a single dimension.
- **Discussion - *Biological interpretation of structure-function coupling and decoupling in aging* (Paragraph 1):** “Structural eigenmodes provide spatial bases constrained by the white-matter connectome, such that low-frequency (smooth) modes represent spatially extended, anatomically supported patterns of activity, whereas high-frequency (rough) modes capture more spatially complex patterns that deviate from structural constraints. Increased coupling therefore reflects a shift of intrinsic functional dynamics toward structurally supported low-frequency modes, while reduced decoupling indicates a diminished contribution from higher-frequency, structurally liberal modes, suggesting reduced spatial complexity and flexibility of intrinsic dynamics.”

2. Can the authors comment on the decision to apply global signal regression in the current analyses? Do similar results emerge if GSR is not applied?

**Response:** We thank the reviewer for raising this important point. We acknowledge that the use of global signal regression (GSR) remains debated in resting-state fMRI preprocessing, as it can reduce global physiological and motion-related fluctuations but may also alter the distribution of functional correlations.

To evaluate the robustness of our findings, we repeated the primary analyses using an otherwise identical preprocessing pipeline without applying GSR. Importantly, the longitudinal effects were virtually identical to those obtained with GSR (Supplementary Table 4), indicating that our coupling/decoupling measures are not driven by the inclusion of GSR.

This pattern is expected given the nature of our metric: the coupling/decoupling indices are derived from graph spectral decomposition of regional BOLD signals with respect to the individual structural connectome, and summarize the relative contributions of low-frequency (structurally aligned/coupled) versus high-frequency (structurally liberal/decoupled) components, rather than correlation structure between regions. Since GSR primarily removes a spatially widespread common-mode component that largely projects onto the smoothest graph modes, it has limited influence on the partitioning between coupled and decoupled components. Together, these results suggest that our conclusions are robust to the use of GSR.

We have added the following descriptions to the revised manuscript:

- **Methods** ***- Image processing*** (**Paragraph 2**): “Global signal regression was applied in the main pipeline to reduce global shared fluctuations. To assess robustness, we repeated the key analyses using the same preprocessing pipeline without GSR.”
- **Results** - ***Network-level structure-function integration showed differential longitudinal changes in older adults*** (the last paragraph): “Sensitivity analyses without GSR yielded longitudinal time effects that were virtually identical to the primary results (Supplementary Table 4), suggesting that our conclusions are robust to the use of GSR.”
- **Supplementary Tables**

**Supplementary Table 4.** Sensitivity analysis without global signal regression (GSR): Age and time effects for structure-function coupling and decoupling in main dataset.

| **Effect** | **Signal type** | **Network** | **80% variance** | | | **90% variance** | | **95% variance** | |
| --- | --- | --- | --- | --- | --- | --- | --- | --- | --- |
|  |  |  | Estimate | | *p* | Estimate | *p* | Estimate | *p* |
| **Time** | Coupled | ECN-A | -0.16 | 0.472 | | -0.23 | 0.214 | -0.17 | 0.356 |
|  |  | ECN-B | -0.09 | 0.656 | | -0.06 | 0.752 | -0.15 | 0.431 |
|  |  | **ECN-C** | **1.16** | **0.001*** | | **0.83** | **0.020*** | **0.82** | **0.018*** |
|  |  | **DMN-A** | **1.46** | **<0.001*** | | **1.28** | **<0.001*** | **1.39** | **<0.001*** |
|  |  | DMN-B | 0.01 | 0.960 | | -0.21 | 0.298 | -0.17 | 0.428 |
|  |  | DMN-C | 0.16 | 0.656 | | 0.06 | 0.854 | 0.09 | 0.806 |
|  |  | **SN-A** | **-0.54** | **<0.001*** | | **-0.45** | **0.013*** | **-0.34** | **0.027*** |
|  |  | SN-B | -0.36 | 0.073 | | -0.24 | 0.217 | -0.19 | 0.292 |
|  | De-coupled | ECN-A | -0.02 | 0.918 | | 0.09 | 0.589 | 0.07 | 0.684 |
|  |  | ECN-B | 0.20 | 0.150 | | 0.12 | 0.418 | 0.15 | 0.377 |
|  |  | **ECN-C** | **-1.04** | **0.002*** | | **-1.00** | **0.005*** | **-0.93** | **0.009*** |
|  |  | **DMN-A** | **-1.12** | **<0.001*** | | **-1.25** | **<0.001*** | **-1.40** | **<0.001*** |
|  |  | DMN-B | 0.27 | 0.192 | | 0.25 | 0.254 | 0.35 | 0.091 |
|  |  | DMN-C | 0.38 | 0.109 | | 0.38 | 0.132 | 0.46 | 0.086 |
|  |  | **SN-A** | **0.38** | **0.035*** | | **0.47** | **0.008*** | **0.43** | **0.008*** |
|  |  | SN-B | 0.18 | 0.182 | | 0.19 | 0.147 | 0.22 | 0.111 |
| **Age** | Coupled | **ECN-A** | **-0.32** | **0.010*** | | **-0.36** | **0.003*** | **-0.34** | **0.007*** |
|  |  | ECN-B | 0.14 | 0.284 | | 0.14 | 0.250 | 0.08 | 0.482 |
|  |  | ECN-C | 0.28 | 0.123 | | 0.31 | 0.067 | 0.30 | 0.085 |
|  |  | DMN-A | 0.23 | 0.124 | | 0.27 | 0.032* | 0.24 | 0.061 |
|  |  | DMN-B | 0.19 | 0.085 | | 0.20 | 0.051 | 0.14 | 0.151 |
|  |  | DMN-C | 0.29 | 0.112 | | 0.26 | 0.124 | 0.14 | 0.439 |
|  |  | SN-A | 0.12 | 0.174 | | 0.05 | 0.607 | 0.12 | 0.224 |
|  |  | SN-B | 0.04 | 0.615 | | 0.10 | 0.224 | 0.11 | 0.121 |
|  | De-coupled | **ECN-A** | **0.23** | **0.030*** | | **0.23** | **0.029*** | **0.28** | **0.005*** |
|  |  | ECN-B | -0.01 | 0.932 | | -0.04 | 0.719 | -0.01 | 0.913 |
|  |  | ECN-C | -0.20 | 0.227 | | -0.22 | 0.187 | -0.23 | 0.176 |
|  |  | DMN-A | -0.23 | 0.054 | | -0.27 | 0.025* | -0.32 | 0.009* |
|  |  | DMN-B | -0.13 | 0.164 | | -0.13 | 0.162 | -0.07 | 0.473 |
|  |  | DMN-C | -0.01 | 0.896 | | -0.02 | 0.863 | -0.09 | 0.428 |
|  |  | SN-A | -0.06 | 0.422 | | -0.03 | 0.658 | -0.02 | 0.778 |
|  |  | SN-B | -0.06 | 0.335 | | -0.07 | 0.264 | -0.13 | 0.031* |

Estimates (*β*) for effects of age and time on structure-function coupling and decoupling from linear mixed modelling in main dataset, controlled for age, gender and education. Estimates are included for all networks of interest. Asterisks (*) indicate significant estimates with uncorrected *p* < 0.05. Networks in bold show estimates that were found to be significant at all three levels of variances.

3. The authors may want to reference another relevant study of cognitive flexibility (TMT) and brain dynamics:

Brain Dynamics Underlying Cognitive Flexibility Across the Lifespan. Kupis L, Goodman ZT, Kornfeld S, Hoang S, Romero C, Dirks B, Dehoney J, Chang C, Spreng RN, Nomi JS, Uddin LQ. Cereb Cortex. 2021 Oct 1;31(11):5263-5274.

**Response**: We thank the reviewer for this helpful suggestion. We have now cited this work in the revised Introduction:

- **Introduction** (Paragraph 4): “In parallel, prior work has demonstrated that cognitive flexibility across the lifespan is supported by large-scale brain dynamics [72]. However, how the functional dynamics constrained by the underlying structural connectome change in aging remains largely unexplored.”

**Reviewer #3:** This manuscript presents a sophisticated longitudinal analysis of how the relationship between brain structure and function changes with age and relates to cognitive flexibility. The authors provide evidence that the Salience Network (specifically the SN-A subnetwork) exhibits a distinct pattern of decreasing alignment and increasing liberality with age, and that these changes are associated with a decline in cognitive flexibility. This finding is novel and has significant implications for understanding the neural underpinnings of cognitive aging.

The manuscript is generally well-written, the methods are rigorous, and the conclusions are mostly supported by the data. The study's key strengths include its longitudinal design, the use of two independent datasets for discovery and validation, and the application of a novel graph signal processing technique (graph Fourier transform) to quantify structure-function integration (alignment and liberality). Nonetheless, I have several comments and suggestions that may help further improve the manuscript.

1. The authors correctly note that only the DMN-A effects survive strict Bonferroni correction across all subnetwork tests. However, for findings that are reproducible across the primary and validation datasets—even if not surviving such strict correction—it may still be meaningful to interpret them cautiously. The authors could also consider applying a less conservative correction method (e.g., False Discovery Rate, FDR) for the primary analysis of the three core networks (ECN, DMN, SN) and their subnetworks. Reporting both uncorrected and FDR-corrected p-values would provide a more nuanced perspective on the results.

**Response**: We thank the reviewer for this thoughtful suggestion. To provide a more nuanced assessment of the statistical evidence, we have additionally applied False Discovery Rate (FDR) correction in the revised manuscript.

Specifically, because our primary hypotheses focused on the subnetworks of three core networks (ECN, DMN, and SN), we performed FDR correction across the full set of subnetwork-level tests, i.e., 8 subnetworks × 2 structure–function indices (coupling and decoupling) = 16 comparisons. We now report uncorrected p-values and FDR-adjusted p-values, alongside Bonferroni-corrected results, in the updated Supplementary Tables 1 and 2. Importantly, the overall pattern of results and conclusions remained largely consistent after FDR correction. Accordingly, we revised the Methods and Results sections to describe this additional correction approach:

- **Methods - *Estimation of aging-related changes in structure-function integration in older adults*** (Paragraph 1): “In addition to Bonferroni correction, we also applied FDR correction across the set of subnetwork-level tests within the ECN, DMN, and SN (8 subnetworks × 2 measures) to provide a complementary, less conservative control of multiple comparisons.”
- **Results** - ***Network-level structure-function integration showed differential longitudinal changes in older adults*** (Paragraph 3): “In addition to Bonferroni-corrected p-values, uncorrected and FDR-adjusted p-values are also reported for subnetwork analyses (see Supplementary Tables 1 and 2).”
- **Supplementary Tables:** Supplementary Tables 1 and 2 have been updated to include uncorrected, FDR-adjusted, and Bonferroni-corrected p-values.

Related to this suggestion, we also conducted an additional analysis examining ROI-level aging effects within the three core networks (see **Response 7 to Reviewer #1**). The ROI-level patterns were broadly consistent with the subnetwork-level findings, with the strongest aging-related coupling/decoupling changes observed in SN-A and DMN-A regions after Bonferroni correction, further supporting the robustness of our findings.

2. Although the authors focus primarily on the three core cognitive networks, it would be valuable to examine age-related changes in structure-function coupling within sensorimotor networks as well. Reporting these results as a control analysis in the Supplementary Information could help contextualize the specificity of the observed effects in higher-order networks.

**Response:** We thank the reviewer for this thoughtful suggestion. We agree that examining sensorimotor systems can provide an informative control analysis to contextualize the specificity of the effects observed in higher-order cognitive networks. Following this recommendation, we performed a control analysis by extending our longitudinal analyses (95% variance) to include the somatomotor and visual networks in addition to the ECN, DMN, and SN subnetworks (10 networks in total). These results are now reported in **Supplementary Table 5**.

Overall, after applying FDR and Bonferroni corrections, the sensorimotor systems did not exhibit significant longitudinal changes in structure–function coupling or decoupling. In contrast, the strongest effects in SN-A and DMN-A, and the effect in ECN-C, remained significant after correction, supporting robust longitudinal alterations in higher-order cognitive and salience/attention-related systems. Together, these control analyses provide additional evidence that the most prominent coupling/decoupling changes are relatively specific to higher-order networks rather than reflecting a global shift across all functional systems.

We have revised the manuscript accordingly:

- **Methods** ***- Derivation of structure-function coupling and decoupling*** (Paragraph 8): “As a control analysis to contextualize the specificity of effects observed in higher-order cognitive networks, we extended the longitudinal analyses (95% variance) to include the somatomotor and visual networks in addition to the ECN, DMN, and SN subnetworks.”
- **Results *- Network-level structure-function integration showed differential longitudinal changes in older adults*** (Paragraph 7): “In a control analysis extending the subnetwork-level longitudinal models to include somatomotor and visual networks (in addition to the ECN, DMN, and SN subnetworks), we found that after applying FDR and Bonferroni corrections, the sensorimotor systems did not exhibit significant longitudinal changes in structure–function coupling or decoupling, whereas SN-A, DMN-A, and ECN-C remained significant after correction, indicating robust longitudinal effects. These control analyses provide additional context supporting the relative specificity of the strongest coupling/decoupling alterations in higher-order cognitive and salience/attention-related systems (Supplementary Table 5).”
- **Supplementary Tables**

**Supplementary Table 5.** Time effects for structure-function coupling and decoupling in main dataset (including sensorimotor networks).

| **Effect** | **Signal type** | **Network** | Estimate | *Raw p* | *FDR-p* | *Bonferroni-p* |
| --- | --- | --- | --- | --- | --- | --- |
| **Time** | Coupled | ECN-A | -0.17 | 0.356 | 0.512 | 1.000 |
|  |  | ECN-B | -0.15 | 0.431 | 0.512 | 1.000 |
|  |  | ECN-C | 0.82 | 0.018* | 0.056 | 0.339 |
|  |  | **DMN-A** | **1.39** | **<0.001*** | **<0.001*** | **<0.001*** |
|  |  | DMN-B | -0.17 | 0.428 | 0.512 | 1.000 |
|  |  | DMN-C | 0.09 | 0.806 | 0.806 | 1.000 |
|  |  | SN-A | -0.34 | 0.027* | 0.073 | 0.509 |
|  |  | SN-B | -0.19 | 0.292 | 0.463 | 1.000 |
|  |  | SomMot | 0.45 | 0.039***** | 0.093 | 0.741 |
|  |  | Visual | 0.07 | 0.617 | 0.690 | 1.000 |
|  | Decoupled | ECN-A | 0.07 | 0.684 | 0.722 | 1.000 |
|  |  | ECN-B | 0.15 | 0.377 | 0.512 | 1.000 |
|  |  | **ECN-C** | **-0.93** | **0.009*** | **0.041*** | 0.164 |
|  |  | **DMN-A** | **-1.40** | **<0.001*** | **<0.001*** | **<0.001*** |
|  |  | DMN-B | 0.35 | 0.091 | 0.172 | 1.000 |
|  |  | DMN-C | 0.46 | 0.086 | 0.172 | 1.000 |
|  |  | **SN-A** | **0.43** | **0.008*** | **0.041*** | 0.155 |
|  |  | SN-B | 0.22 | 0.111 | 0.192 | 1.000 |
|  |  | SomMot | -0.43 | 0.016***** | 0.056 | 0.300 |
|  |  | Visual | -0.33 | 0.356 | 0.512 | 1.000 |

Estimates (*β*) for effects of time on structure-function coupling and decoupling from linear mixed modelling in main dataset, controlled for age, gender and education. Estimates are included for all networks of interest. Asterisks (*) indicate significant estimates with uncorrected *p* < 0.05. Networks in bold show estimates that were found to be significant after FDR or Bonferroni correction.

3. While the present study focuses on structure-function coupling, it would be informative to first report age-related changes in functional and structural networks separately before presenting the coupling results. Including these analyses in the Supplementary Information would provide a more comprehensive background and help readers interpret the coupling findings.

**Response:** We thank the reviewer for this helpful suggestion. We agree that reporting age-related changes in functional and structural measures separately can provide useful background for interpreting the structure–function integration results.

Using the same longitudinal dataset, our prior work (cited in the manuscript; <https://www.sciencedirect.com/science/article/abs/pii/S1053811916002342>; <https://onlinelibrary.wiley.com/doi/abs/10.1002/hbm.24036>) has reported progressive age-related changes in functional organization, including reduced within-network functional specialization in higher-order systems (e.g., ECN and DMN) and altered ECN–DMN segregation trajectories that relate to processing-speed decline. In parallel, structural connectivity in normal aging is characterized by a widespread, progressive decline in white matter integrity, with preferential vulnerability of highly connected hub regions within higher-order networks (e.g., DMN) and long-range white matter connections (<https://www.sciencedirect.com/science/article/pii/S1053811923004214>). Building on these established findings, the current study extends this line of work by explicitly quantifying how structural connectivity constrains intrinsic functional dynamics (BOLD activity) within the three core cognitive networks via graph-spectral coupling/decoupling indices.

To directly address the reviewer’s concern within the current manuscript, we added a supplementary control analysis examining longitudinal effects on functional BOLD activity. Specifically, we computed the mean intrinsic BOLD activity within each ECN, DMN, and SN subnetwork, and applied the same linear mixed-effects models to test time- and age-related effects. These results are provided in **Supplementary Table 6**. Briefly, only intrinsic BOLD activity in DMN-B and SN-A showed significant longitudinal change, with opposite directions (DMN-B increasing and SN-A decreasing over time). These functional changes could plausibly contribute to the observed structure–function integration effects, alongside the well-documented decline in long-range white matter connectivity. Notably, the comparatively modest effects were observed for these conventional component measures (uncorrected p < 0.05). When relating longitudinal changes in intrinsic BOLD activity in DMN-B and SN-A to corresponding changes in cognitive flexibility, no significant associations were observed (p = 0.058 and p = 0.18, respectively). Taken together, structure–function integration indices may provide a more sensitive marker of aging-related changes supporting cognitive flexibility.

We have revised the manuscript accordingly:

- **Methods -** ***Estimation of aging-related changes in structure-function integration in older adults* (Paragraph 4)**: “To provide background for interpreting structure–function integration results, we additionally computed mean intrinsic BOLD activity within each ECN, DMN, and SN subnetwork and tested longitudinal effects using the same linear mixed-effects models, and related the longitudinal changes in intrinsic subnetwork BOLD activity to corresponding changes in cognitive flexibility.”
- **Results -** ***Network-level structure-function integration showed differential longitudinal changes in older adults* (Paragraph 8)**: “In a control analysis examining longitudinal changes in mean structural connectivity and intrinsic BOLD activity within ECN, DMN, and SN subnetworks (Supplementary Table 6), only intrinsic BOLD activity in DMN-B and SN-A showed significant longitudinal change, with opposite directions (DMN-B increased, whereas SN-A decreased). When relating longitudinal changes in intrinsic BOLD activity in DMN-B and SN-A to corresponding changes in cognitive flexibility, no significant associations were observed (p = 0.058 and p = 0.18, respectively). Compared with these modest component-level changes, structure–function integration indices showed stronger longitudinal effects, suggesting higher sensitivity to aging-related change in this dataset.”
- **Discussion** (**Paragraph 1**): “Age-related alterations in FC and SC have been widely reported, including reduced structural connectivity integrity/efficiency and reduced functional segregation across large-scale systems. Our graph-spectral coupling/decoupling metrics are not intended to replicate these established findings; rather, they provide complementary information by quantifying how the structural connectome constrains intrinsic functional dynamics. In this way, coupling/decoupling indices may offer an additional and potentially more sensitive perspective on aging-related reorganization beyond conventional FC or SC measures alone.”
- **Supplementary Tables**

**Supplementary Table 6.** Age and time effects for BOLD activity in main dataset.

| **Effect** | **Network** | **Estimate** | ***p*** | **Effect** | **Network** | **Estimate** | ***p*** |
| --- | --- | --- | --- | --- | --- | --- | --- |
| **Time** | ECN-A | 0.0033 | 0.197 | **Age** | ECN-A | 0.0019 | 0.207 |
|  | ECN-B | 0.0034 | 0.339 |  | ECN-B | 0.0004 | 0.826 |
|  | ECN-C | 0.0000 | 0.225 |  | ECN-C | 0.0000 | 0.925 |
|  | DMN-A | 0.0000 | 0.703 |  | DMN-A | 0.0000 | 0.362 |
|  | DMN-B | 0.2446 | 0.011* |  | DMN-B | 0.0957 | 0.109 |
|  | DMN-C | -0.0196 | 0.313 |  | DMN-C | -0.0001 | 0.809 |
|  | SN-A | -0.0570 | 0.025* |  | SN-A | 0.0028 | 0.166 |
|  | SN-B | -0.0075 | 0.172 |  | SN-B | -0.0003 | 0.718 |

Estimates (*β*) for effects of age and time on BOLD activity from linear mixed modelling in main dataset, controlled for age, gender and education. Estimates are included for all networks of interest. Asterisks (*) indicate significant estimates with uncorrected *p* < 0.05.

4. The observed increase in alignment and decrease in liberality in DMN-A and ECN-C is intriguing but requires further interpretation. The authors suggest this pattern may reflect increased reliance on short-range connections and potentially reduced network efficiency—a plausible explanation, though additional evidence supporting this argument would strengthen the discussion. Furthermore, the concepts of "alignment" and "liberality" are central to the paper but remain somewhat abstract. Although the methodological explanation is clear, a more thorough discussion of the biological interpretation of these metrics would be highly beneficial for the general readership.

**Response:** We thank the reviewer for this insightful comment. We agree that the observed increase in structure–function coupling (alignment) and decrease in decoupling (liberality) in DMN-A and ECN-C requires deeper biological interpretation, and that these terms may remain abstract for readers unfamiliar with the graph-spectral framework. To improve accessibility, we have revised the manuscript to link the original terminology to the more familiar terms “structure–function coupling” and “decoupling” (see also **Response 3 to Reviewer #1**).

In the revised manuscript, we have substantially expanded the **Discussion** to clarify the biological interpretation of these metrics within a low-dimensional network organization framework. Specifically, in the graph-spectral approach, functional activity is decomposed with respect to structural eigenmodes, which can be interpreted as intrinsic spatial bases supported by white-matter architecture. Low-frequency (smooth) modes represent spatially extended activity patterns that are strongly constrained by anatomical connectivity, whereas high-frequency (rough) modes reflect more spatially complex patterns that deviate from structural constraints. Accordingly, increased coupling (alignment) indicates that intrinsic functional dynamics increasingly occupy structurally supported low-frequency modes, while reduced decoupling (liberality) reflects diminished contribution from higher-frequency, structurally liberal modes. We now discuss how this shift can be interpreted as reduced spatial complexity and reduced flexibility of intrinsic functional dynamics.

Importantly, we further grounded this interpretation in recent work on structure–function relationships and network organization. In particular, a recent study in *Nature Communications* (2025) (<https://www.nature.com/articles/s41467-025-65156-6#Sec7> ) demonstrated that structural degeneration is associated with reduced amplitudes of intrinsic functional gradients and altered inter-gradient relationships, consistent with a “network collapse” mechanism characterized by increasing dominance of anatomically constrained activity patterns and loss of flexible, distributed dynamics. We now link our findings in DMN-A to this framework, proposing that increased coupling and reduced decoupling may reflect a similar shift toward structurally dominated, low-dimensional functional organization in aging, particularly in higher-order associative systems.

Regarding the interpretation in terms of short-range connectivity and reduced efficiency, we have revised the Discussion to clearly distinguish empirical observations from mechanistic hypotheses. Rather than asserting reduced efficiency directly, we now frame increased coupling as reflecting greater reliance on structurally supported, locally coherent modes of activity, consistent with prior evidence for preferential vulnerability of long-range integrative processes in aging. We emphasize that this interpretation is mechanistic and indirect, and note that future work combining graph-spectral metrics with explicit measures of connection length, efficiency, or gradient geometry will be important to directly test these hypotheses.

We have revised the manuscript accordingly:

- **Discussion**: “***Biological interpretation of structure-function coupling and decoupling in aging:*** Within the graph-spectral framework, structure–function coupling (alignment) and decoupling (liberality) can be interpreted in relation to low-dimensional bases of brain organization supported by anatomical connectivity. Structural eigenmodes provide spatial bases constrained by the white-matter connectome, such that low-frequency (smooth) modes represent spatially extended, anatomically supported patterns of activity, whereas high-frequency (rough) modes capture more spatially complex patterns that deviate from structural constraints. Increased coupling therefore reflects a shift of intrinsic functional dynamics toward structurally supported low-frequency modes, while reduced decoupling indicates a diminished contribution from higher-frequency, structurally liberal modes, suggesting reduced spatial complexity and flexibility of intrinsic dynamics.

This interpretation is consistent with recent work demonstrating that structural degeneration is associated with reduced amplitudes of intrinsic functional gradients and altered inter-gradient relationships, a pattern interpreted as a form of low-dimensional “network collapse” characterized by increasing dominance of anatomically constrained activity patterns [95]. In this context, the aging-related increase in coupling and decrease in decoupling observed in DMN-A—robust across the main analysis, sensitivity analyses using a finer parcellation resolution, and ROI-level modeling—may reflect a shift toward structurally dominated intrinsic dynamics in higher-order associative systems, which rely on distributed integration and flexible reconfiguration to support executive function.”
• **Discussion** - ***Differential structure-function integration changes in task-positive and task-negative networks in aging*** (Paragraph 2): “While we speculated that this pattern may involve greater reliance on short-range connectivity and reduced network efficiency, we emphasize that this interpretation is indirect. Rather, our findings suggest that aging may bias intrinsic functional dynamics in DMN toward more locally coherent, structurally constrained modes, consistent with reduced flexibility of large-scale network organization. Future work integrating graph-spectral measures with explicit metrics of connection length, efficiency, and functional gradients will be important to directly test these mechanistic hypotheses.”

5. In the Introduction, the authors could better emphasize the significance of their study, particularly its longitudinal design, within the existing literature.

**Response**: We thank the reviewer for this helpful suggestion. We agree that the significance of the present work—particularly the value of its longitudinal design—should be more clearly highlighted in the Introduction (also see **Response 2 to Reviewer #1**).

In the revised manuscript, we have strengthened the framing to emphasize that most prior structure–function integration studies in aging are cross-sectional, making it difficult to distinguish true within-person change from cohort effects, and that our longitudinal approach allows us to quantify within-individual trajectories of structure–function coupling/decoupling and relate these trajectories to longitudinal decline in cognitive flexibility. We also clarify that we assess intrinsic structure–function integration from resting-state fMRI, providing a task-independent characterization of aging-related changes that complements prior task-based work.

We have revised the Introduction accordingly:

- **Introduction** (Paragraph 5): “Since most prior structure–function integration studies in aging are cross-sectional, making it difficult to distinguish true within-person change from cohort effects, our longitudinal approach allows us to quantify within-individual trajectories of structure–function coupling/decoupling. Particularly, we assessed intrinsic structure–function integration from resting-state fMRI, providing a task-independent characterization of aging-related changes that complements prior task-based work.”
